# Liquid biopsy-based monitoring of residual disease in multiple myeloma by analysis of the rearranged immunoglobulin genes–A feasibility study

**DOI:** 10.1371/journal.pone.0285696

**Published:** 2023-05-26

**Authors:** Anita Marx, Magdolna Osváth, Bence Szikora, Orsolya Pipek, István Csabai, Ákos Nagy, Csaba Bödör, Zsolt Matula, Ginette Nagy, András Bors, Ferenc Uher, Gábor Mikala, István Vályi-Nagy, Imre Kacskovics

**Affiliations:** 1 Department of Immunology, Institute of Biology, ELTE Eötvös Loránd University, Budapest, Hungary; 2 Doctoral School of Biology, ELTE Eötvös Loránd University, Budapest, Hungary; 3 Department of Physics of Complex Systems, ELTE Eötvös Loránd University, Budapest, Hungary; 4 Department of Pathology and Experimental Cancer Research, HCEMM-SE Molecular Oncohematology Research Group, Semmelweis University, Budapest, Hungary; 5 National Institute of Hematology and Infectious Diseases, Central Hospital of Southern Pest, Budapest, Hungary; Universita degli Studi di Milano-Bicocca, ITALY

## Abstract

The need for sensitive monitoring of minimal/measurable residual disease (MRD) in multiple myeloma emerged as novel therapies led to deeper responses. Moreover, the potential benefits of blood-based analyses, the so-called liquid biopsy is prompting more and more studies to assess its feasibility. Considering these recent demands, we aimed to optimize a highly sensitive molecular system based on the rearranged immunoglobulin (Ig) genes to monitor MRD from peripheral blood. We analyzed a small group of myeloma patients with the high-risk t(4;14) translocation, using next-generation sequencing of Ig genes and droplet digital PCR of patient-specific Ig heavy chain (IgH) sequences. Moreover, well established monitoring methods such as multiparametric flow cytometry and RT-qPCR of the fusion transcript *IgH*::*MMSET* (IgH and multiple myeloma SET domain-containing protein) were utilized to evaluate the feasibility of these novel molecular tools. Serum measurements of M-protein and free light chains together with the clinical assessment by the treating physician served as routine clinical data. We found significant correlation between our molecular data and clinical parameters, using Spearman correlations. While the comparisons of the Ig-based methods and the other monitoring methods (flow cytometry, qPCR) were not statistically evaluable, we found common trends in their target detection. Regarding longitudinal disease monitoring, the applied methods yielded complementary information thus increasing the reliability of MRD evaluation. We also detected indications of early relapse before clinical signs, although this implication needs further verification in a larger patient cohort.

## Introduction

Multiple myeloma is the hematologic malignancy of the second highest incidence [1], in fact, it is the most common malignancy that resides primarily in the bone marrow. Customarily, monitoring of the disease is done using plasma markers of the characteristic M-protein and free light chains secreted by the malignant plasma cells, as well as by bone marrow examinations, as required. In the recent decade, due to the advent of novel therapies, up to 90% of myeloma patients may reach complete remission [2], therefore an unmet need emerged for novel techniques to quantitatively evaluate low-level disease [3]. Multiparametric flow cytometry (MFC) is a well standardized and accessible method for disease monitoring, which was further improved by novel antibody panels, refined sample preparation methods, automation, and the analysis of much higher number of cells, which all contributed to the increased sensitivity of the so-called next-generation flow (NGF) method [3,4]. NGF, together with next-generation sequencing (NGS) of bone marrow samples are the currently available most sensitive techniques to monitor minimal/measurable residual disease (MRD), reaching tumor detection sensitivities up to $1\times 10^{-6}$ [3–7]. While their sensitivity and prognostic value is on par, NGF has the advantage of standardization, fast turnaround time, availability in a wide range of patients and lower costs. On the other hand, NGS provides a considerable advantage in sample requirements as it can be applied to archived samples as well, in contrast to NGF where fresh sampling is indispensable. Furthermore, NGS provides detailed information on clonal composition, relatedness, and clonal evolution over time. It is also shown by different groups that for reaching the same level of sensitivity, NGS requires less input of cells than NGF, and finally the evaluation of the resulting data may be more user-friendly with the available NGS platforms [4–7]. These two methods albeit having their pros and cons, both proved to be effective in assessing the MRD status of myeloma patients with strong prognostic value [7] and they are both approved by the International Myeloma Working Group (IMWG) [8].

As bone marrow biopsies represent highly invasive and painful examinations and may be misleading if the disease in remission remains focal (may also require confirmation by imaging techniques),”liquid biopsies” of peripheral blood samples that represent the entire body of the myeloma patient offer a possible noninvasive solution, which also allows for frequent disease monitoring [9,10]. Some studies already indicated that spatial heterogeneity, this characteristic feature of multiple myeloma [9,11], can be potentially overcome by liquid biopsy testing which is shown to represent the entire tumor burden of the patients [12,13]. Due to these advantages, there is an increasing interest on the applicability of liquid biopsy, in parallel with advances in bone marrow MRD monitoring. Although in multiple myeloma circulating tumor cells and cell-free tumor DNA may be readily detected at diagnosis [14–17], it is challenging to do so in remission [14,18–20]. Even if the most advanced methods currently may not be sufficient for reaching the same sensitivity as with bone marrow samples [21], several studies already indicated the promise of peripheral blood-based monitoring and characterization of multiple myeloma. These studies applied various molecular approaches, such as whole genome/exome sequencing [20,22], targeted gene panel sequencing [23,24] or targeted amplicon sequencing [18,25] on NGS platforms, or droplet digital PCR [19] or a combination of these techniques [26,27].

**Fig 1:**
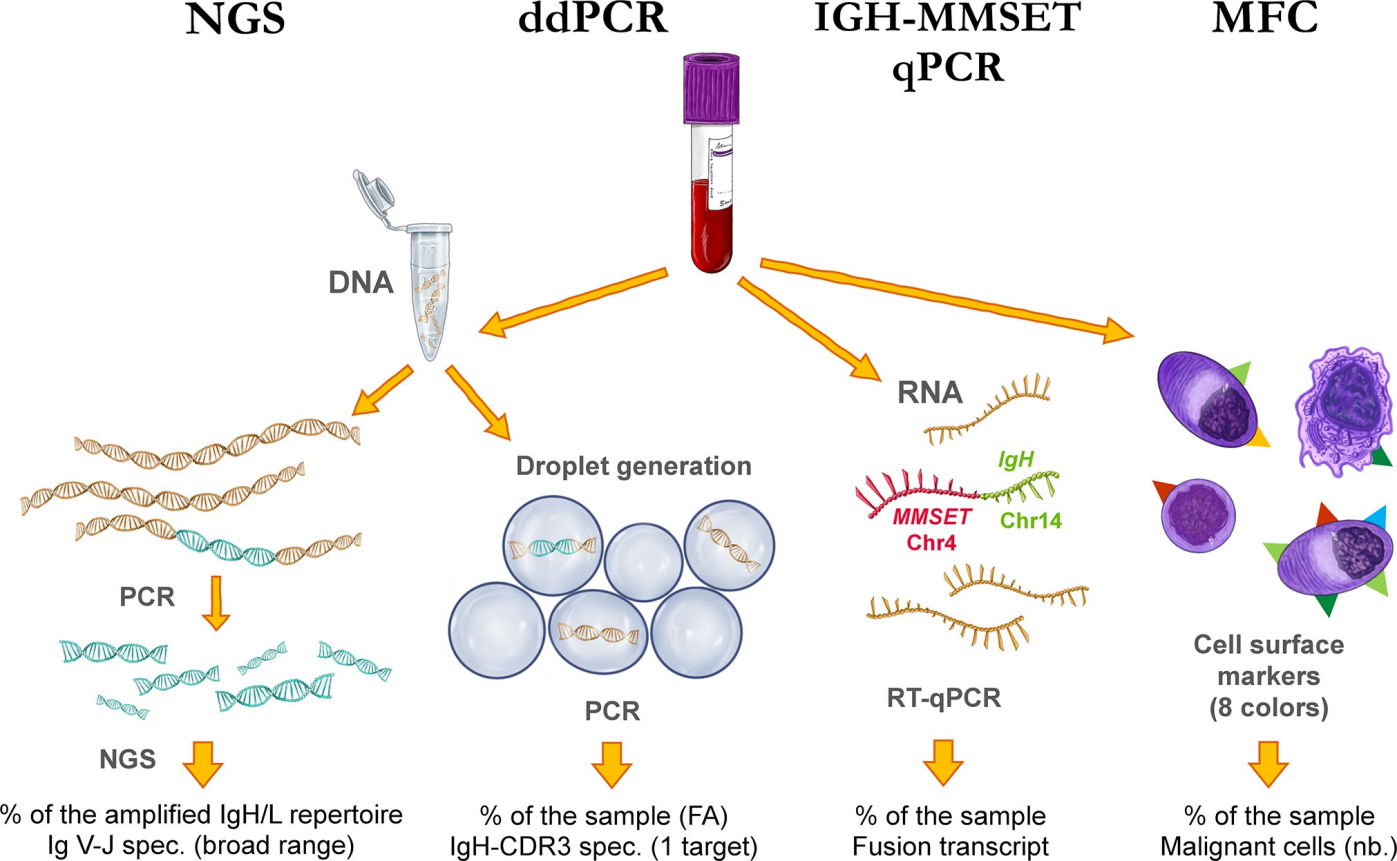
A summary of the methods and their specific approaches to the monitoring of multiple myeloma utilized in this study. NGS = next-generation sequencing, ddPCR = droplet digital PCR, IGH-MMSET qPCR = *IgH*::*MMSET* fusion transcript specific reverse transcription qPCR, MFC = multiparametric flow cytometry, *IgH/L* = immunoglobulin heavy/light chain (respectively), Ig V-J = immunoglobulin V and J gene segments, CDR3 = complementarity determining region 3, FA = fractional abundance, *MMSET* = multiple myeloma SET domain-containing protein (also called *NSD2* = nuclear SET domain-containing protein 2), Chr = chromosome.

In our study, we concentrated on a special high-risk subgroup who takes up around 15% of myeloma patients, those with translocation t(4;14) [28–30] and developed alternative means to detect and measure disease burden using peripheral blood. Flow cytometry, next generation sequencing, droplet digital PCR (ddPCR), and *IgH*::*MMSET* translocation-specific reverse-transcription qPCR were used to detect circulating tumor cells (all four methods) and tumor specific cell-free DNA (NGS, ddPCR), and compared their sensitivity, usefulness, and correlation with actual myeloma cell burden. Each method analyzes a different aspect of the myeloma patients’ disease. Multiparametric flow cytometry utilizes a holistic view of cell surface markers in order to identify tumor-specific phenotypes and monitor the disease either from bone marrow or peripheral blood. The translocation-specific qPCR relies on the specificity of this myeloma subgroup, the unique fusion transcript of *IgH* and *MMSET* (multiple myeloma SET domain-containing protein) [31] and uses the presence of this marker to follow the disease course. Finally, both our NGS and ddPCR methods are based on the patient-specific myeloma clone’s rearranged V(D)J (variable-diversity-joining) sequence of the immunoglobulin (Ig) genes (Fig 1). The main principle of ddPCR is the sequestration of the sample into nanoliter-size droplets (0.7–0.8 nl, [32]), which not only enables the analysis of fragmented DNA with a potentially greater accuracy, but also allows for a direct quantitative analysis. On the other hand, the NGS analyses allow for a more holistic view of the Ig repertoire by the amplification and sequencing of millions of different rearranged Ig genes from the sample, potentially highlighting subclonal processes besides the target-based monitoring. Of note, the ddPCR has the technical advantage in the need of less input template for the analyses, making it a more available method for generally low concentration cell-free DNA (cfDNA) samples, thus allowing for a wider range of analyzable samples. These two techniques take advantage of the terminally differentiated B-cell stage of the myeloma cells, as they are no longer subject to Ig gene rearrangement or somatic hypermutation. These Ig genes were shown to be stable throughout the disease course and multiple sub-clonal evolution events in myeloma progression [33–35]. Originating from single B-cells activated during a primary immune response, each plasma cell bears unique Ig sequences, and as myeloma develops at this terminally differentiated stage of B-cell development, the Ig heavy and light chain CDR3 (complementarity determining region 3) sequences are prone to be potential biomarkers for disease monitoring, as shown by several groups in the past years [3,6,7,18,25,36–43].

## Materials and methods

### Patients and samples

11 multiple myeloma patients were initially enrolled in our study harboring high-risk t(4;14) translocation, each with a diverse course of treatment. Due to their aggressive disease characteristics, 3 patients had to be excluded from further analyses. Among them, in case of one patient, no dominant myeloma clone sequence was found in the diagnostic bone marrow sample, possibly due to the extramedullary nature of this patient’s disease, so our successful target identification rate was 90.9% (10/11). In addition, we were unable to collect follow-up samples from two other patients because of their early passing (their target sequences can be found in Table E in S1 File). Thus, the remaining 8 patients were monitored at various intervals with a median follow-up time of 25 months (ranging from 9 months to 111 months). Detailed clinical data are provided in the S1 Table.

**Fig 2:**
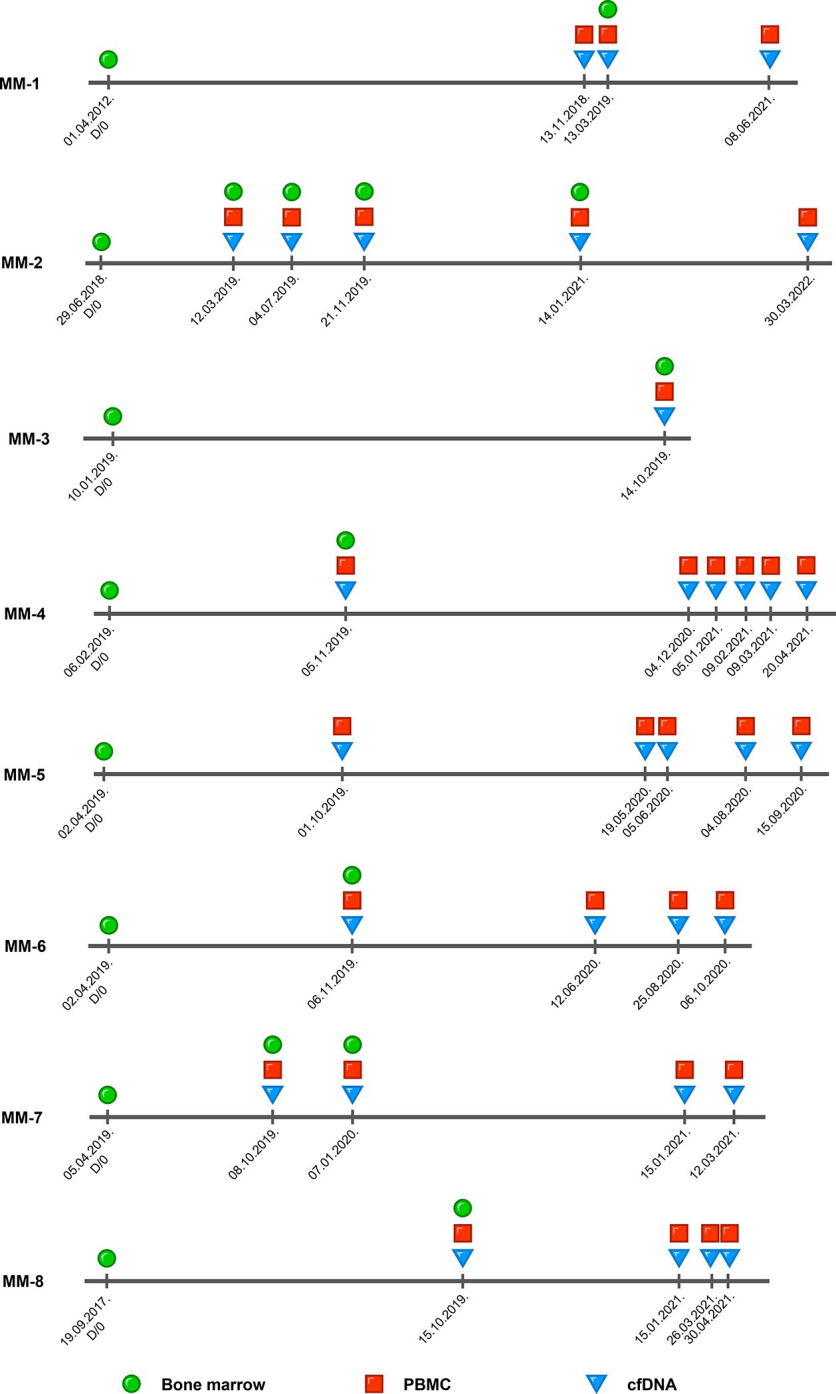
Schematic overview of each patient’s follow-up schedule with the acquired sample types. The diagnostic / target defining timepoint is indicated as ‘D/0’. Bone marrow samples are marked with a green circle, peripheral blood mononuclear cell samples with a reddish square, and cell-free DNA samples with a blue triangle over each monitoring timepoint. PBMC = peripheral blood mononuclear cells, cfDNA = cell-free DNA.

The samples were collected in the Central Hospital of Southern Pest, National Institute of Hematology and Infectious Diseases, Budapest, Hungary. Bone marrow samples were collected by sternal bone marrow puncture after the patients’ written consent. Bone marrow and peripheral blood mononuclear cells (10^6^−10^7^ cells) and plasma (4–7 ml) were isolated using Ficoll-Paque PLUS (GE Healthcare) density gradient centrifugation within a few hours after sample collection. The isolated samples were stored at -80°C until DNA extraction. The total number of analyzed follow-up samples is listed in Tables B and C in S1 File. A schematic overview of each patient’s sampling timepoints with sample types are presented on Fig 2, where ‘D/0’ marks the diagnostic timepoint.

The study was conducted in accordance with the Declaration of Helsinki and approved by the Ethics and Scientific Committee of the Central Hospital of Southern Pest, National Institute of Hematology and Infectious Diseases with permission of the National Institute of Pharmacy and Nutrition (OGYÉI/50268-8/2017). Written informed consent was obtained from all subjects involved in the study.

### DNA extraction for Ig V(D)J-based analyses

Bone marrow mononuclear cells (BM-MNC) and peripheral blood mononuclear cells (PBMC) were subjected to genomic DNA (gDNA) isolation using the DNeasy Blood&Tissue Kit (Qiagen), following the manufacturer’s protocol. DNA concentrations were measured by Multiskan Sky spectrophotometer with Microdrop Plate (Thermo Scientific). The PBMC samples were not enriched prior to DNA isolation, the circulating tumor cells were assessed at the molecular level later with our bioinformatics pipeline.

Circulating cell-free DNA (cfDNA) was isolated from the plasma samples with the QIAamp Circulating Nucleic Acid Kit (Qiagen), following the manufacturer’s protocol. The cfDNA concentrations were measured by Qubit4 fluorometer with dsDNA HS Assay Kit (Thermo Scientific), following the manufacturer’s protocol.

### NGS protocol

#### Multiplex PCRs targeting the immunoglobulin genes

For the immunoglobulin heavy chain specific reactions, the heavy chain FR1 (framework region 1) BIOMED-2 primer set was used, while the light chain specific reactions were carried out with the mixture of the kappa and the lambda light chain BIOMED-2 primer sets [44]. For the reactions, 2 pmol/μl each forward mix and 10 pmol/μl each reverse mix was added, the primer sequences can be found in the original publication [44]. We used 500 ng of gDNA and 25 ng of cfDNA when possible. The PCRs had a touchdown heat profile designed for multiplex PCRs in a SureCycler 8800 (Agilent) instrument, the cycling conditions can be found in the S1 File: Methods.

Following agarose gel electrophoresis, the heavy chain FR1 specific PCR products were expected around 300–350 bp, while the light chain specific products were expected in a wider range, around 150–200 bp and 300 bp [44]. The appropriate PCR products were extracted and subjected to clean-up using the NucleoSpin Gel&PCR Clean-up Kit (Macherey-Nagel), following the manufacturer’s protocol to retrieve the amplicons.

#### Library construction and sequencing

Library preparation and sequencing of the amplicons were carried out at the Genomic Medicine and Bioinformatics Core Facility, Department of Biochemistry and Molecular Biology, University of Debrecen, Debrecen, Hungary. For library preparation, the Ovation Ultralow Library System v2 (Tecan) was used, following the manufacturer’s protocol. For an optimal sequencing depth, 20 ±1–2 samples per NGS run were analyzed. The NGS was carried out on an Illumina MiSeq instrument with the MiSeq Reagent Kit v2 (Illumina) using paired-end chemistry.

#### Bioinformatics analysis pipeline

The resulting FASTQ files were shared via the Illumina BaseSpace cloud repository. For data processing, our university’s cloud server system (Kooplex Hub) with custom written codes in Python were used. For quality control, a preprocessing code was applied which filters out short read pairs in which the sequence of the reverse primer could not be detected or in which the average Phred base quality score (Q-score) did not reach 20, separately for the two reads of the read pair (Q20). This Q-score filtering also alleviates the possibility of false positive detection in the subsequent analysis. The remaining read pairs were exported into a FASTA file (per each sample) which was uploaded to the IMGT/HighV-QUEST tool [45–47] for immunologically relevant evaluation. The results were further analyzed by our custom codes, which determined the number and percent of productive immunoglobulin sequences in each sample and calculated the incidence of the myeloma target sequences from these data. These target sequences were initially identified from the NGS analysis of diagnostic bone marrow samples, as sequences supported by the largest number of reads in the analyzed repertoire, based on the high tumor infiltration of the bone marrow at diagnosis. For reliable assessment, myeloma target sequences were defined in cases when the difference in the number of supporting reads between the most abundant and the second most frequent sequence was at least one order of magnitude (e.g., thousands vs. hundreds), and supporting reads comprised at least 5% of the whole repertoire. We hypothesized that the presence of a highly clonal sequence from bone marrow at diagnosis is linked to the myeloma, since the same aspirate samples were used for myeloma diagnosis and risk stratification before our analyses. Peripheral blood samples from follow-up timepoints were then analyzed with the objective of recovering the previously identified myeloma target sequences. The incidence of a target sequence (expressed in percentages) was calculated from the number of productive sequence read copies.

For the monitoring sample analyses (primarily from liquid biopsy), an empirical threshold for NGS target positivity was defined according to the following. A sample was considered NGS positive when the target was found at an abundance of at least 0.001%. We concluded that detection under this threshold usually meant only 1–2 copies which could be a background noise signal based on off-target detections. The latter refers to the target sequence occurrences in independent patient samples, in cases when cross-sample contamination can be ruled out. Thus, in addition to the Q20 filtering, applying this empirical threshold to NGS data can further alleviate the background effect of our measurements.

### ddPCR protocol

Custom ddPCR assays were designed to target the patient specific IgH CDR3 sequences (for technical details and the assay sequences, see Table A in S1 File). To make our ddPCR analyses more robust and reliable, an *RPP30* copy number assay (dHsaCP1000485, Bio-Rad) was used as a reference gene assay in multiplex setting. For the reaction mixes, a 1:1 ratio of IgH-CDR3 target specific FAM assay (PrimePCR Custom Assay, Bio-Rad) and *RPP30* reference HEX assay was added. When possible, 25 ng of template DNA was added. For negative sample control, a 1:1 volume mix of 5 healthy individuals’ PBMC gDNA was prepared. The QX200 Automated Droplet Generator (Bio-Rad) was used for droplet generation, following the manufacturer’s protocol with the recommended reagents and labware. The PCR was carried out in a C1000 Touch Thermal Cycler (Bio-Rad), the heat profile can be found in the S1 File: Methods. For data acquisition, the QX200 Droplet Reader (Bio-Rad) was used, controlled by the QuantaSoft software (version 1.7, Bio-Rad). Results were considered reliable if >10 000 droplets per sample were detected (total event number), these were evaluated using the QuantaSoft software.

### Multiparametric flow cytometry measurements

For the flow cytometry analysis, EDTA anticoagulated samples were used. The bone marrow samples were strained using special tubes (5 ml polystyrene round-bottom tube with cell-strainer cap, cat.no:352235, BD Biosciences). For the staining of the cells (BM-MNC or PBMC), the following markers were used, according to the manufacturers protocol: CD19 PC7 (cat. no.: BCI-F-IM3628, Beckman Coulter Company), CD20 APC-H7, (cat.no.: 561172, BD Biosciences), CD27 PerCP-Cy5.5 (cat.no.: 560612, BD Biosciences), CD28, PerCP-Cy5.5 (cat.no.: 337181, BD Biosciences), CD38 APC (cat.no.: 345807, BD Biosciences), CD45 Krome Orange (cat.no.: BCI-F-IM36294, Beckman Coulter Company), CD56 FITC (cat. no:345811, BD Biosciences), CD81 APC-H7 (cat. no:656647, BD Biosciences), CD117 PE, (cat. no.: 332785, BD Biosciences), CD138 VioBlue (cat.no.:130-119-843, Miltenyi Biotec), Kappa FITC (cat. no.:349516, BD Biosciences) (intracellular staining), Lambda PE (cat. no.:349516, BD Biosciences) (intracellular staining). For the intracellular staining, BD Intrasure Kit was used (cat.no.: 641778 BD Biosciences). The acquisition of the samples was done on a FACS Canto II cytometer (BD Biosciences), and the analyses were done with the Infinicyt (ver.2.0.2c, Cytognos S.L.) software.

### IGH-MMSET qPCR measurements

Total RNA was isolated from bone marrow and/or from peripheral blood samples by routine TRIzol (Invitrogen) method. Besides routine bone marrow measurements, total RNA from whole blood served as liquid biopsy containing circulating plasma cells and potentially circulating myeloma cells. Reverse transcription was performed by High-Capacity cDNA Reverse Transcription Kit (Life Technologies) using random hexa primers.

qPCR measurements for *IgH*::*MMSET* mRNA detection were performed on a LightCycler 480 II (Roche) instrument. Reactions were performed using LightCycler 480 Probes Master kit (Roche), with 300 nM of each primer and 200 nM of probe and 240 ng cDNA per well. The sequences are available upon request. The PCR program can be found in the S1 File: Methods. The resulting *IgH*::*MMSET* fusion mRNA quantities were compared to the mRNA expression levels of each sample’s *ABL1* housekeeping gene using the deltaCt method, and it was expressed as percentages in the Results section.

### Statistics

Analysis of correlation between different methods were carried out in GraphPad Prism 5 (version 5.03 for Windows, GraphPad Software, San Diego California USA) using Spearman correlation test with a significance level of P ≤ 0.05. To account for multiple testing, Bonferroni correction was used.

## Results

### Ig-NGS and data analysis–technical details

By analyzing around 20 samples per sequencing run, an average of 832 272 raw Ig heavy chain sequences were obtained per sample, ranging from 295 713 to 1 738 340. The Ig light chain data yielded 788 705 raw sequences per sample in average, ranging from 252 092 to 1 786 781. The mean proportion of reads passing the Q20 prefiltering analysis were 90% and 89% in case of heavy and light chain data, respectively, indicating the high quality of the sequencing runs. The percentages of productive immunoglobulin sequences were calculated based on the IMGT analyses, where the gDNA samples yielded an average of 84% and 62% for the heavy and light chain, respectively; while the cfDNA samples had 81% and 34% productive sequences per heavy and light chain, respectively. This measure indicates the prevalence of rearranged immunoglobulin sequences in our samples, hence the plasma samples’ lower values (especially in case of the light chain). It is also worth noting that the light chain analyses omitted the Jκ-Cκ intron-RSS and the Kde primers.

The analysis of the light chain amplicons was carried out from the 150–200 bp fraction, as we found that the diagnostic bone marrow samples mainly produced PCR products in that range. In two cases we found equal or stronger bands in the 300 bp range in the diagnostic bone marrow sample, which was analyzed separately. Patient MM-2 had a same intensity band in both light chain ranges, but only the 150–200 bp fraction contained a highly clonal sequence, the 300 bp product was oligoclonal. In case of patient MM-5 the majority of the PCR products were around 300 bp (S3 Fig), and the NGS analysis showed that although both fractions contained the same highly clonal sequence, the 300 bp fragments in a much greater abundance. Thus, in this case the 300 bp fragments were analyzed as well for all follow-up samples, we evaluated both size ranges’ results and found that the 300 bp amplicons mirrored the other measures better, so these data were used for further analyses. Of note, the 150–200 bp data also showed the same trends in target detection as the 300 bp data but with much lower target values (S3 Table: Abbreviations and Notes tab). This phenomenon may be explained by the BIOMED-2 primer distributions as only patient MM-5 had *IGKV2* gene which produces longer amplicons with the appropriate primers than the other patients’ *IGK* or *IGL* genes [44].

### Myeloma-specific target identification by NGS

Archived gDNA from the patients’ diagnostic bone marrow samples were analyzed, and we identified their immunoglobulin heavy chain and light chain myeloma clone sequences which were used as target sequences for monitoring with NGS and ddPCR (CDR3-specific assay designs based on the heavy chain sequences). Of note, patient MM-4’s diagnostic sample proved to be unusual and only a heavy chain target was identified; details on that analysis can be found in the S1 File: Methods and S1 File: Results. The patients’ identified target sequences and their respective V-and J-genes can be found in Table 1.

**Table 1:** The identified heavy and light chain myeloma targets.

Patient ID	CDR3 sequence (aa)	V gene	J gene
MM-1	CGGDGRSDHPDYW	Homsap *IGHV3-23*	Homsap *IGHJ4*
	CQQYSAYPWTF	Homsap *IGKV1-5*	Homsap *IGKJ1* or Homsap *IGKJ4*
MM-2	CASNLYETSGHYYDGDYYGMDVW	Homsap *IGHV1-18*	Homsap *IGHJ6*
	CSSYTSASNTLELVF	Homsap *IGLV2-14* or Homsap *IGLV2-18*	Homsap *IGLJ2* or Homsap *IGLJ3*
MM-3	CASHLSFGESYYFGMDVW	Homsap *IGHV3-21*	Homsap *IGHJ6*
	CQQYGSSPPYTF	Homsap *IGKV3-20* or Homsap *IGKV3D-20*	Homsap *IGKJ2*
MM-4	CARDLGNKAFEDW	Homsap *IGHV1-2*	Homsap *IGHJ3*
MM-5	CVRCEYGSRSFPHLAFNIW	Homsap *IGHV5-51*	Homsap *IGHJ3*
	CMQGTHWPYTF	Homsap *IGKV2-30* or Homsap *IGKV2D-30*	Homsap *IGKJ2*
MM-6	CARKNYYFDSW	Homsap *IGHV1-69*	Homsap *IGHJ4*
	CQQYESYSALTF	Homsap *IGKV1-5*	Homsap *IGKJ4*
MM-7	CTSGPGLGAFDIW	Homsap *IGHV3-21*	Homsap *IGHJ3*
	CQSADSSGSFYVVF	Homsap *IGLV3-25*	Homsap *IGLJ2* or Homsap *IGLJ3*
MM-8	CARIEKLRSSSLYAFDYW	Homsap *IGHV2-70*	Homsap *IGHJ4*
	CQQSYSTLYTF	Homsap *IGKV1-39* or Homsap *IGKV1D-39*	Homsap *IGKJ2*

Amino acid (aa) CDR3 sequences together with their respective V/J genes, heavy chain (*IGHV-IGHJ*) and light chain (*IGKV-IGKJ* or *IGLV-IGLJ*). The possible alleles are listed in Table D in S1 File. Data from the IMGT-HighV/QUEST analysis.

### Correlation of the applied methods with the clinical standard M-protein and FLC measurements

We compared our data obtained by five different methods (IgH NGS, ddPCR, IgL NGS, IGH-MMSET qPCR, and MFC) to the clinical standard measurement of M-protein. These analyses contain data pairs from all available patients and all timepoints, serving as a holistic comparison of our methods (Table 2). To assess the effect of sample type, we analyzed the V(D)J-based methods’ data separately per sample type: bone marrow (BM) sample, PBMC or cfDNA. Correlation analyses were also carried out on combined data sets per each V(D)J method: liquid biopsy samples or all samples from one method.

**Table 2:** Spearman correlation analyses of our methods with M-protein data.

M-protein vs.	Number of data pairs	Spearman r	95% confidence interval	P value	Adjusted P value
BM NGS-H	19	0.8082	0.5491 to 0.9255	<0.0001	<0.0017
PB NGS-H	30	0.6296	0.3384 to 0.8107	0.0002	0.0034
CF NGS-H	22	0.763	0.4933 to 0.8989	<0.0001	<0.0017
BM ddPCR	19	0.9021	0.7526 to 0.9632	<0.0001	<0.0017
PB ddPCR	30	0.6986	0.4432 to 0.8491	<0.0001	<0.0017
CF ddPCR	29	0.6483	0.3597 to 0.8237	0.0001	0.0017
BM NGS-L	17	0.8305	0.5718 to 0.9389	<0.0001	<0.0017
PB NGS-L	24	0.7044	0.4099 to 0.8659	0.0001	0.0017
CF NGS-L	24	0.7136	0.4252 to 0.8704	<0.0001	<0.0017
IGH-MMSET	12	0.8379	0.4940 to 0.9551	0.0007	0.0119
MFC	8	0.8625	-	0.0107	0.1819
LB NGS-H	52	0.6804	0.4941 to 0.8069	<0.0001	<0.0017
LB ddPCR	60	0.6514	0.4702 to 0.7799	<0.0001	<0.0017
LB NGS-L	48	0.7218	0.5445 to 0.8374	<0.0001	<0.0017
NGS-H	71	0.6452	0.4795 to 0.7665	<0.0001	<0.0017
ddPCR	79	0.6651	0.5156 to 0.7753	<0.0001	<0.0017
NGS-L	65	0.6285	0.4483 to 0.7596	<0.0001	<0.0017

We compared the different sample types per NGS or ddPCR separately to the M-protein data, as well as the liquid biopsy samples together, and finally the summary of each molecular method. Default significance thresholds of the GraphPad Prism 5 software were used (significant correlation from P ≤ 0.05). To correct for multiple comparisons, Bonferroni correction was used. BM = bone marrow sample, PB = PBMC sample, CF = cfDNA sample, NGS-H = Ig heavy chain NGS, ddPCR = droplet digital PCR, NGS-L = Ig light chain NGS, IGH-MMSET = *IgH*::*MMSET* fusion transcript specific RT-qPCR, MFC = multiparametric flow cytometry, LB = Liquid Biopsy (PBMC and cfDNA samples together).

Despite the limited patient cohort and sample size, we found significant correlation among all our methods with M-protein. We noticed slightly higher r values in case of the BM samples, probably due to the fact that we only analyzed BM samples at diagnosis and usually at the first monitoring timepoint–the diagnostic sample always had the highest tumor burden measured with each method, while the m1 timepoint is after the usually successful first line therapy where the disease intensity is low. The combined analysis of liquid biopsy samples did not differ greatly from the individual sample type-wise results, similarly to the combination of all sample types per V(D)J method. Both NGSs and the ddPCR showed similar correlations to the M-protein, while methods with the least data pairs like IGH-MMSET qPCR and MFC had slightly higher r values. It is worth noting that MFC had less degree of significance, and the adjusted P value indicated a non-significant correlation, probably due to the lack of available data. Some illustrative dot plots from these MFC measurements are detailed in the S1 File and shown as S1 and S2 hin.

We also compared our Ig light chain NGS (NGS-L) and MFC results to the free light chain (FLC) measurements where sufficient data was available (Table 3) in the same manner as with the M-protein data.

**Table 3:** Spearman correlation analyses of our relevant methods with FLC data.

FLC vs.	Number of data pairs	Spearman r	95% confidence interval	P value	Adjusted P value
BM NGS-L	11	0.8545	0.5073 to 0.9631	0.0008	0.0048
PB NGS-L	13	0.5912	0.04116 to 0.8662	0.0334	0.2004
CF NGS-L	13	0.6217	0.08928 to 0.8778	0.0233	0.1398
MFC	6	0.6375	-	0.1733	1.0398
LB NGS-L	26	0.6183	0.2925 to 0.8154	0.0008	0.0048
NGS-L	37	0.6488	0.4029 to 0.8074	<0.0001	<0.0006

We compared the different sample types per NGS-L separately to the FLC data, as well as the liquid biopsy samples together, and finally the summary of this method. Default significance thresholds of the GraphPad Prism 5 software were used (significant correlation from P ≤ 0.05). To correct for multiple comparisons, Bonferroni correction was used. FLC = free light chain, BM = bone marrow sample, PB = PBMC sample, CF = cfDNA sample, NGS-L = Ig light chain NGS, MFC = multiparametric flow cytometry, LB = Liquid Biopsy (PBMC and cfDNA samples together).

The analysis of combined sample types like liquid biopsy or the overall NGS-L method versus FLC values yielded an improved significance in their correlation. With the exception of MFC, all compared methods showed various degrees of correlation to FLC, although when considering the adjusted P values, only the combined analyses and the BM samples were significant.

Despite the low sample numbers and small patient cohort, we obtained significant correlations of our methods to the gold-standard M-protein, and the liquid biopsy-based molecular methods to the FLC data.

### Multi-method disease monitoring

In order to test whether the aforementioned methods may be suitable for monitoring the disease course of multiple myeloma from blood, we summarized our results obtained with these methods into patient-wise follow-up graphs, accompanied by their respective clinical data. The raw data from each method can be found in the S3 Table.

In our limited-sized patient cohort (n = 8), we identified 3 distinct scenarios according to disease course and response to therapy seen by our methods. These patients had various treatment regiments, and although most of them received autologous stem cell transplant (ASCT), their disease outcomes could be categorized into the following scenarios. Patients who reached sufficient response to therapy and remained responsive during our study (MM-2, MM-3) were termed ‘responsive’. The other two groups are intertwined in respect of therapy response. One of them features weak response to therapy and fast progression (MM-5, MM-6, termed ‘rapid refractory’), while the other group had at least once a sufficient response according to our methods, but despite the therapy they eventually relapsed during the study (MM-1, MM-4, MM-7, MM-8), termed ‘relapsing’. In case of this latter group, we had the opportunity to assess the (early) signs of their relapse.

**Fig 3:**
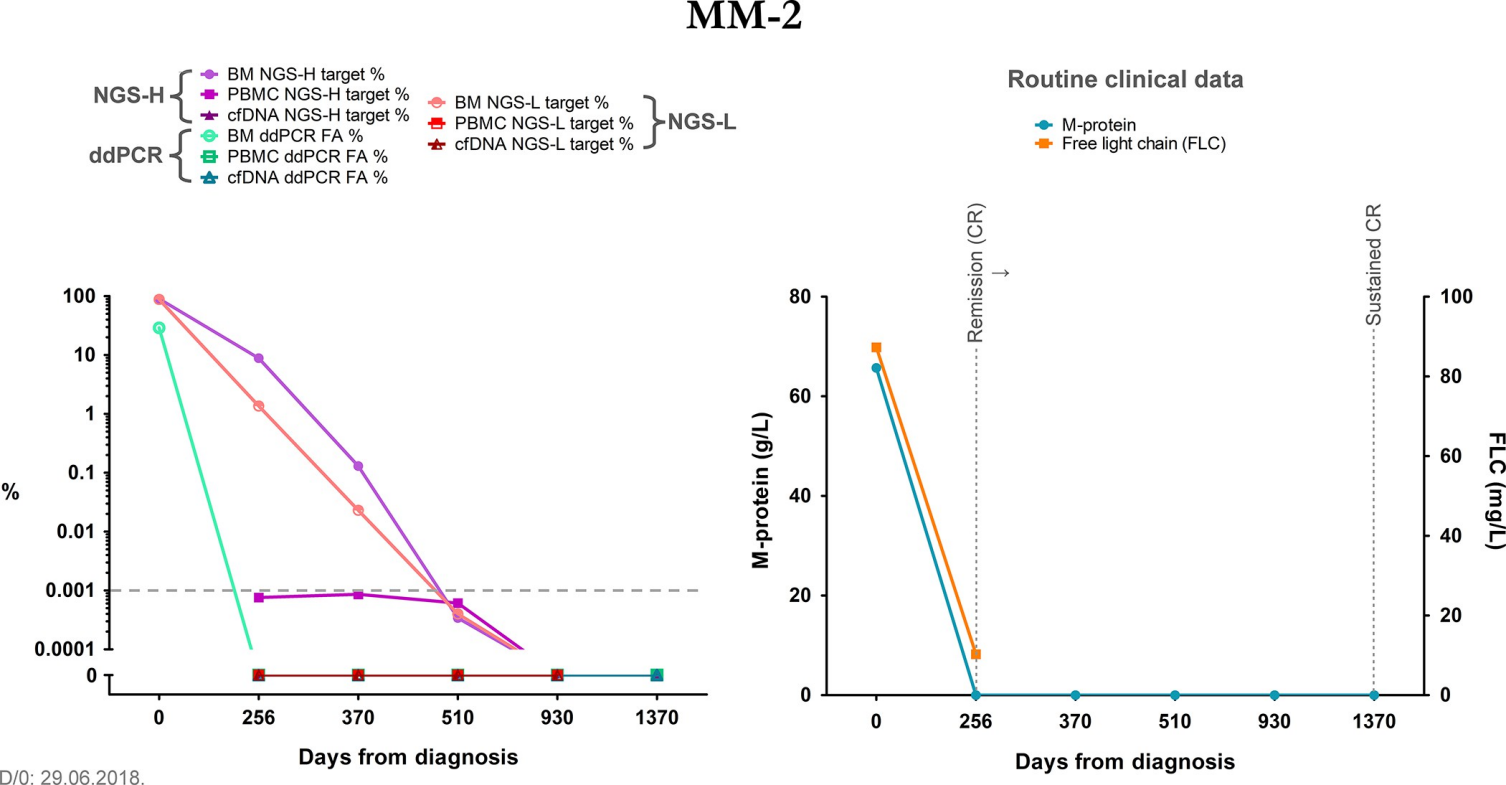
Summary graph of patient MM-2, representing the responsive patient group. The right graph summarizes relevant clinical data, the left graph contains the results of our methods at each monitoring timepoint, the latter are indicated as days from diagnosis on both graphs. The grey dashed line indicates the empirical threshold of NGS-positive results. The y-axis depicts the log10 of the data from our methods; additionally, it was broken to indicate 0 values for better visualization. NGS-H and NGS-L = Ig heavy chain specific and Ig light chain specific NGS (respectively), ddPCR = droplet digital PCR, FA = fractional abundance, BM = bone marrow, PBMC = peripheral blood mononuclear cells, cfDNA = cell-free DNA, D/0 = diagnosis, FLC = free light chain, CR = complete response.

The responsive patient group showed on-threshold target occurrence or even absence in their monitoring liquid biopsy samples during our study. For example, in case of patient MM-2, although these detections were just under the empirical threshold from the first follow-up timepoint onward, by day 930 all our molecular methods indicated the absence of the target, thus we could observe the diminution of myeloma burden at the molecular level. Where bone marrow samples were also accessible, we found a gradual decrease and elimination of the target from the bone marrow as well. These trends were also supported by the clinical data like disease staging and M-protein/FLC measurements (Fig 3). These patients had maintained their CR (complete response) status after their last monitoring timepoints.

**Fig 4:**
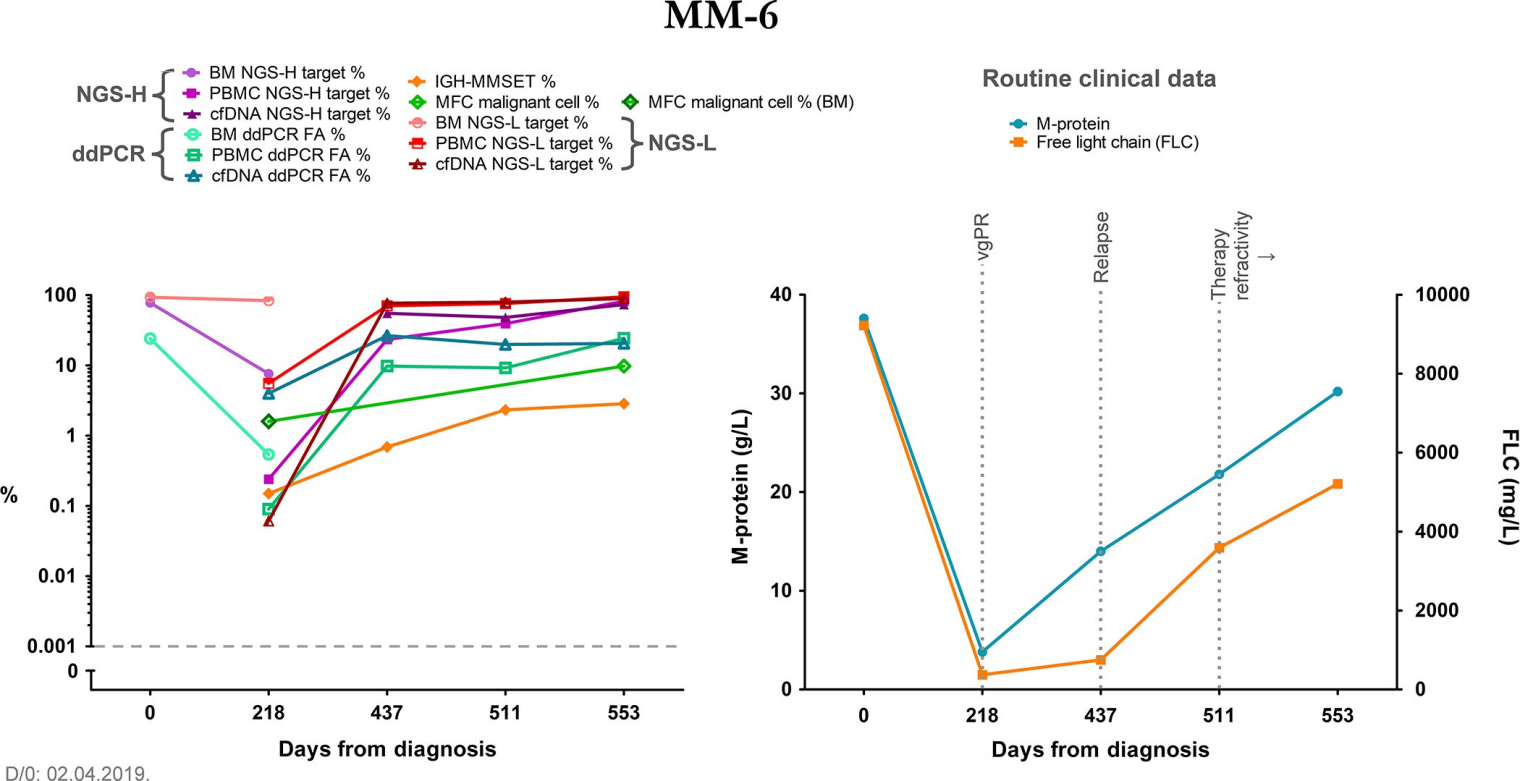
Summary graph of patient MM-6, representing the rapid refractory patient group. The right graph summarizes relevant clinical data, the left graph contains the results of our methods at each monitoring timepoint, the latter are indicated as days from diagnosis on both graphs. These data did not go under the empirical threshold of NGS-positive results, so the grey dashed line is only shown to uniformize the graphs. The y-axis depicts the log10 of the data from our methods; additionally, it was broken to indicate 0 values for better visualization–in this case it was done only to uniformize the graphs. NGS-H and NGS-L = Ig heavy chain specific and Ig light chain specific NGS (respectively), ddPCR = droplet digital PCR, FA = fractional abundance, BM = bone marrow, PBMC = peripheral blood mononuclear cells, cfDNA = cell-free DNA, IGH-MMSET = *IgH*::*MMSET* fusion transcript specific RT-qPCR, MFC = multiparametric flow cytometry, D/0 = diagnosis, FLC = free light chain, vgPR = very good partial response. MFC measurement from the bone marrow is indicated with a darker green frame of the respective data point.

The rapid refractory group featured patients with high tumor burden and elevated target presence in liquid biopsy samples also at earlier monitoring timepoints (with partial response) with at least two different methods. During our study, their targets became highly prevalent in liquid biopsy samples with several methods, in parallel with clinically proven disease progression and relapse (Fig 4). These patients passed away soon after their last monitoring timepoint.

**Fig 5:**
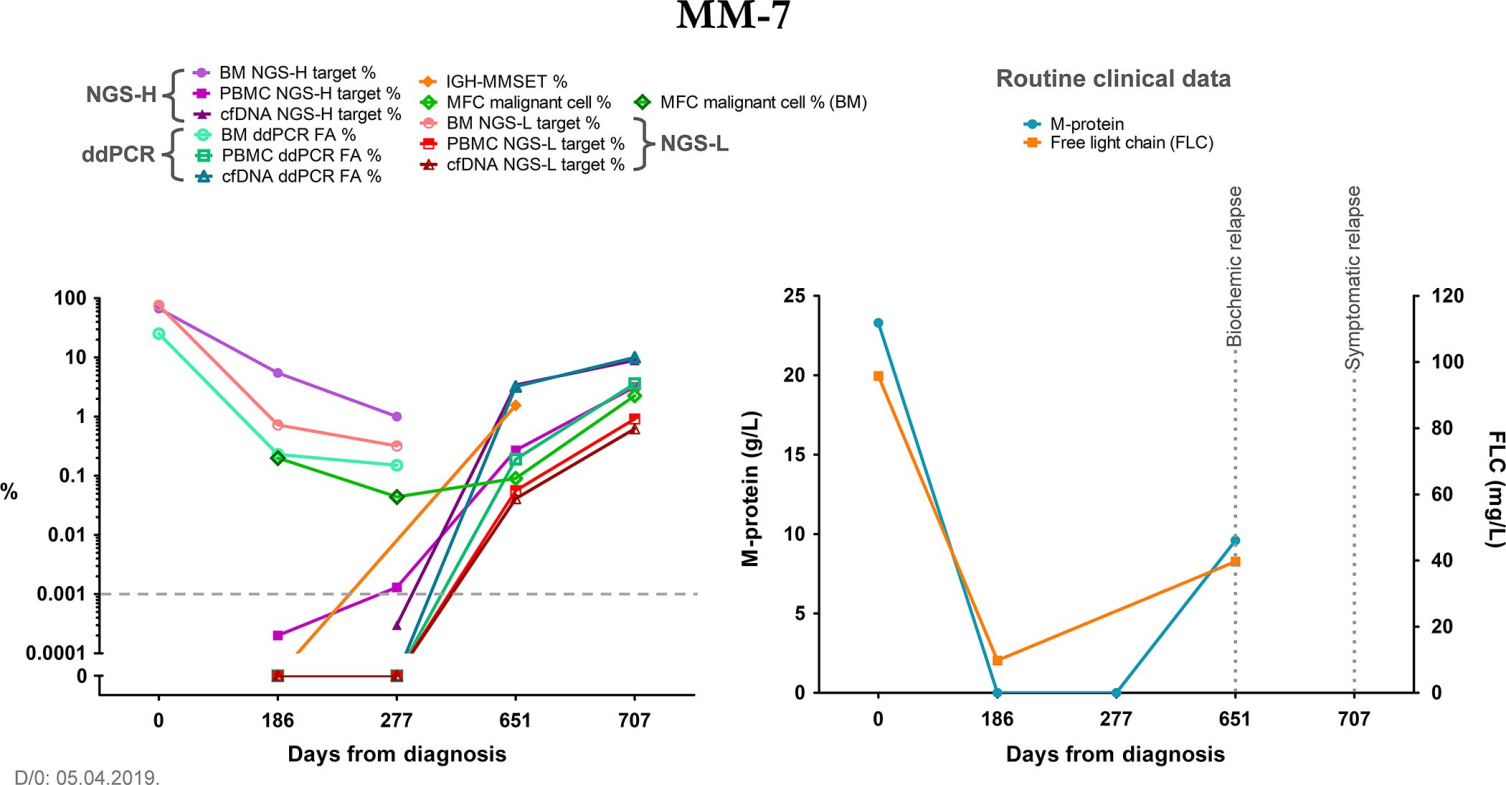
Summary graph of patient MM-7, representing the relapsing patient group. The right graph summarizes relevant clinical data, the left graph contains the results of our methods at each monitoring timepoint, the latter are indicated as days from diagnosis on both graphs. The grey dashed line indicates the empirical threshold of NGS-positive results. The y-axis depicts the log10 of the data from our methods; additionally, it was broken to indicate 0 values for better visualization. NGS-H and NGS-L = Ig heavy chain specific and Ig light chain specific NGS (respectively), ddPCR = droplet digital PCR, FA = fractional abundance, BM = bone marrow, PBMC = peripheral blood mononuclear cells, cfDNA = cell-free DNA, IGH-MMSET = *IgH*::*MMSET* fusion transcript specific RT-qPCR, MFC = multiparametric flow cytometry, D/0 = diagnosis, FLC = free light chain. MFC measurement from the bone marrow is indicated with a darker green frame of the respective data points.

**Fig 6:**
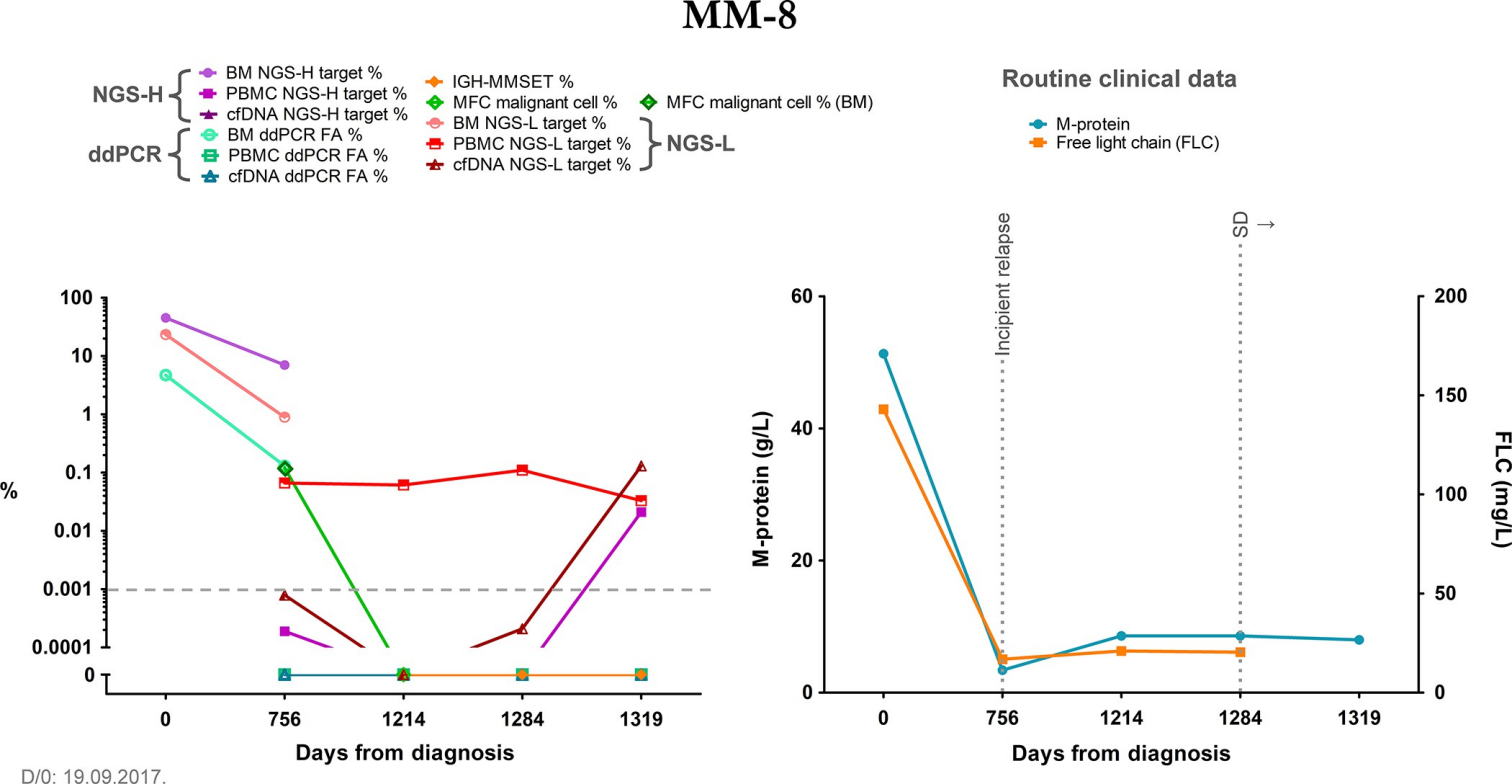
Summary graph of patient MM-8 from the relapsing patient group, further providing a valuable insight into disease monitoring and early relapse detection. The patient relapsed two months after the last follow-up timepoint. The right graph summarizes relevant clinical data, the left graph contains the results of our methods at each monitoring timepoint, the latter are indicated as days from diagnosis on both graphs. The grey dashed line indicates the empirical threshold of NGS-positive results. The y-axis depicts the log10 of the data from our methods; additionally, it was broken to indicate 0 values for better visualization. NGS-H and NGS-L = Ig heavy chain specific and Ig light chain specific NGS (respectively), ddPCR = droplet digital PCR, FA = fractional abundance, BM = bone marrow, PBMC = peripheral blood mononuclear cells, cfDNA = cell-free DNA, IGH-MMSET = *IgH*::*MMSET* fusion transcript specific RT-qPCR, MFC = multiparametric flow cytometry, D/0 = diagnosis, FLC = free light chain, SD = stable disease. MFC measurement from the bone marrow is indicated with a darker green frame of the respective data point.

The third, relapsing patient group included the most patients from our study, featuring a stage of therapy response and subsequent relapse of their disease during our study. Although this response phase varied greatly between patients from remission to just reduced disease activity or stable disease, it always preceded the evident recurrence of the myeloma. Due to these features, this patient group was ideal for assessing our methods in relapse detection. In general, the cfDNA results with either method were more dynamic and seemingly mirrored the disease stages better (Figs 5 and 6). In two cases we found indication that at least one of our methods detected a rising in target detection rates before other clinical measures suggested a relapse. In the case of patient MM-4, we detected the presence of the target with the NGS-H and ddPCR systems from the cfDNA sample at the m2 follow-up date, one month before the incipient relapse at the m3 timepoint. From these approaches, ddPCR of the cfDNA samples remained consistently positive throughout our study. Of note, IGH-MMSET qPCR measurements were available from the m3 timepoint, which indicated the presence of myeloma from peripheral blood throughout our study, also predating the confirmed relapse at the m5 timepoint (data in S3 Table, not visualized). In the other case, with patient MM-8, we detected the target sequence in PBMC samples by the NGS-L approach from the first monitoring timepoint onward, with a consistent presence throughout our study. From the cfDNA samples, we noticed the emergence of the target at the m3 follow-up timepoint (although under the empirical threshold) and the subsequent rapid increase at the m4 timepoint. At this latter timepoint, we also detected the NGS-H target from the PBMC sample (Fig 6). These data all predated the relapse of this patient by two months. As our timeframe was restricted to monthly monitoring, these findings indicate the detection of increase in tumor burden at least a month earlier. One of these patients passed away soon after the last monitoring timepoint, while the others showed various disease statuses.

## Discussion

Recently, treatment-fee remission supported by regular evaluation of minimal residual disease has become standard of care in the treatment of chronic myelogenous leukemia [48–50]. With respect to multiple myeloma, studies incorporating MRD assessment into treatment decision-making, such as the recently published MASTER trial projects the utility and the need for sensitive monitoring techniques [2]. In this study, our patient cohort harboring the high-risk t(4;14) translocation was selected due to the more aggressive nature of their myeloma and the usually accelerated disease course [51,52], which emphasizes the need for a dynamic and sensitive additional monitoring method. The availability of a RT-qPCR based molecular monitoring method based on the characteristic fusion transcript of this translocation was also tempting. To achieve frequent sampling and thus closer monitoring of therapy efficiency, liquid biopsy is an attractive tool by overcoming the painful bone marrow sampling of these already fragile patients. Moreover, bone marrow sampling is prone to spatial bias due to the possible patchy localization and/or uneven presence of the myeloma in the body, which can lead to false assessments of the actual tumor burden, especially in the case of macrofocal disease [9,11]. Several groups indicated that the presence of myeloma can be detected in liquid biopsy [12,13,16–20,22–25,27,36,53–59], which correlates with routine clinics and disease status [12,19,20,22,53,54,57]. Although these correlations and their clinical value are not always clear [55], it is already established that detecting minimal/measurable residual disease is a strong indicator of progression-free survival and overall survival [6,7,10,21,24,25,37–41,51,59–62] as per the IMWG criteria, which determined a cut-off of 10^−5^ as MRD negativity in the bone marrow, as a universally attainable compromise [8]. To evaluate the feasibility of liquid biopsy in multiple myeloma, our group aimed at testing different highly sensitive methods on multiple myeloma patients and compared them to already established methods. We designed our analysis considering the use of widely accessible resources, e.g., the BIOMED-2 primer sets instead of the ClonoSeq service for the NGS, as well as a MFC setup in lieu of NGF protocols–of note, we only had access to these methods at the time of our study. Despite these technological limitations and the relatively low number of patients, we found promising results using these techniques which correlated well with one another. Moreover, by the enrollment of patients outside the frameworks of any clinical study, we were able to assess these methods and their feasibility in a ‘real-word’ study, irrespective of treatment regimens, in everyday situations.

As any other method used in the clinical context, these molecular approaches also have their limits, which mainly stem from the availability, quantity, and quality of the samples. In our system, a first line NGS of the diagnostic bone marrow is indispensable for the monitoring of the patient’s disease, as this is the target-defining step where we can identify the myeloma’s immunoglobulin heavy and light chain sequences, with emphasis on the complementarity determining region 3 (CDR3), which is the most variable portion of the immunoglobulin sequences generated somatically by the V(D)J rearrangement process during B-cell development and maturation. Due to the particular characteristics of this process, each mature B-cell and their terminally differentiated descendants, the plasma cells, encode exactly one pair of unique immunoglobulin heavy chain and light chain [63,64]. As multiple myeloma cells are malignantly transformed postgerminal lymphoid cells, they also carry unique immunoglobulin sequences [5,64], that is shown to be largely stable during the course of this disease [33–35], unlike most mutated or driver genes which are subject to intense (sub)clonal evolution [11,26,34,53,65], which even increases due to treatment pressure [20,66]. Thus, it is crucial for our analysis to identify the myeloma specific CDR3 sequence of each patient. In our cohort, we found a high success rate of myeloma clone identification, with only one patient lacking amplifiable clonal immunoglobulin sequences from the diagnostic bone marrow sample. In this case, patient MM-290 had extramedullary disease at the time of sampling, which may have prevented our system to identify a target for monitoring. Thus, adequate bone marrow sampling of the baseline disease is crucial for these studies. This phenomenon and limitations were already noted in other groups’ works [12,13,20,22,26,59] and in the FDA guidance to the use of MRD [67], which highlights this possible drawback of bone marrow sampling. However, in case of proper sample acquisition, the baseline target detection with NGS presents no additional difficulties, as the detection sensitivity highly exceeds the potentially lowest baseline tumor burden of the bone marrow, which needs to be in a defined percentage of infiltration by clonal plasma cells to be characterized as multiple myeloma per the IMWG updated diagnostic criteria [68]. This diagnostic tumor abundance can be translated to the molecular level, so the myeloma immunoglobulin sequence can easily be identified. Of note, we could successfully define the target sequences (IgH, IgL kappa) of patient MM-1 even from an archived bone marrow sample at the early, smoldering myeloma phase.

The analysis of liquid biopsy usually means the study of circulating cell-free nucleic acids (DNA or RNA), but the term can also be applied to the circulating cells [9,58,69], more precisely the circulating tumor/myeloma cells in this context. In our study we used NGS and ddPCR for analyzing different portions of the liquid biopsy, namely cell-free DNA (cfDNA) and peripheral blood mononuclear cells (PBMC), as the latter contains the circulating myeloma cells (being a malignant plasma cell). To assess feasibility of these methods, we compared the results to IGH-MMSET qPCR and MFC data, as well as clinical measures.

Even though proper statistical comparison was not possible due to the low number of data pairs, we found trends that suggested similar target detection between IGH-MMSET qPCR and our molecular methods, for example the gradual increase of the target in patient MM-6 (Fig 4), or the temporary negativity and then disease progression in patient MM-7 (Fig 5). We found the same trends with MFC and the V(D)J-based measurements, where the few bone marrow data pairs coincided well, and also the liquid biopsy samples showed similar trends with each method. When compared to the clinical routine M-protein levels, we found statistically significant correlations with all of our methods, detailed in the Results section “Correlation of the applied methods with the clinical standard M-protein and FLC measurements”, which accentuates the potential applicability of these V(D)J-based methods besides the already established and standardized monitoring tools (IGH-MMSET qPCR and MFC). In one case, with patient MM-8, we found discrepancy with our NGS-L data of PBMC and all the other measurements, indicating the presence of the myeloma despite the negativity with every other method and the low levels of the clinical routine M-protein and FLC. At the last monitoring timepoint, we detected the target from the cfDNA sample as well (NGS-L), together with the re-emergence of the NGS-H target in the PBMC sample. These data predated the clinical relapse of this patient by 2 months. Taken together, we were able to detect potential signs of early relapse in this patient, which indicates a promising utilization of these methods in therapy follow-up.

Evidently, the armada of these methods are not always feasible to perform in every clinical setting, we should be able to choose the right tool to complement the routine measurements according to each situation. The choice is to be based on the disease characteristics of the patient and the principles of each method, and the availability and nature of the sample. For example, when bone marrow sampling is inevitable (due to other evaluations), the additional analysis by NGS may be more remunerative, while when only blood samples are available, ddPCR measurements of the cfDNA fraction can be preferred. To illustrate this assumption, we can regard the principles of these methods detailed at the end of the Introduction and in Fig 1. With our established ddPCR system, the results suggested the possible advantage of cfDNA over gDNA in target detection, although not significantly. When comparing Ig heavy chain NGS (NGS-H) and ddPCR (which targets the IgH CDR3 of the patients), we got similar results with similar trends, moreover these data coincided with the other methods and clinical information, further increasing their reliability. In general, we found that cfDNA sample analysis with both ddPCR and NGS yielded a more dynamic measure of the disease burden, following the disease status and its changes closely, which was already indicated by several groups [9,18,24,57,59].

The analysis of the Ig light chain locus by NGS (NGS-L) provided an independent and complementing measurement to the previously detailed heavy chain-based methods, as in some cases we detected the presence of the myeloma even when the heavy chain data implied negativity (see patient MM-8 on Fig 6). That phenomenon might be due to the lower diversity of the light chain rearrangements as they lack the D segment from their CDR3, thus the target sequence could be detected from a less diverse background [42]. This same feature leads to the reduced specificity of the light chain CDR3, as we found more prevalent “off-target detection” with the NGS-L analyses. This means that in cases when cross-sample contamination could be ruled out, we detected traces of target sequences in independent patients’ samples, which was more characteristic for NGS-L than for NGS-H. The same issue was present at our early ddPCR assay designs to light chain CDR3, when we obtained high background signals, hence only heavy chain CDR3 specific ddPCR measurements were utilized for analysis.

From these off-target detection rates, we determined an empirical threshold for NGS positivity of the samples (see Materials and Methods section “Bioinformatics analysis pipeline”), depicted by a gray dashed line on the left graphs of Figs 3–6. NGS-H or NGS-L data just below this empirical threshold were termed as on-threshold NGS detection. When considering samples with this on-threshold detection, we found that they showed changes (disappearance or positivity) during monitoring according to the patient groups (responsive or relapsing, respectively), or they stayed on threshold before their eventual change, which suggests that despite their lower reliability, on-threshold NGS detections may be taken into account as they follow the disease course. To further investigate the possible role of these data, additional experiments are needed as they can improve the sensitivity of our system.

As it was indicated earlier in the literature, the sensitivity of single gene targeted sequencing may not be sufficient for reliable disease monitoring [41,42,55]. To increase the detection sensitivity, one can utilize multiple genetic targets, which complement each other [10,58,70,71], as it can be seen in our study when combining the Ig heavy and light chain analyses. Another approach can be the use of different methods with the same target, as we did with the NGS-H and ddPCR. Of course, all different methods can be combined to multiple targets, so these data can reinforce each other while being complementary, allowing for a more precise disease follow-up.

With the availability of bone marrow samples at least in the first monitoring timepoints, we could assess the patients’ response to therapy in both liquid biopsy and bone marrow. In case of patient MM-2, we were able to collect bone marrow samples from almost all monitoring timepoints, and we could follow the gradual elimination of the target sequence from the patient. In all cases we found higher detection rates in bone marrow samples than in liquid biopsy, which is supported by earlier findings of several research groups [4,17,55,72]. This is partly due to the inherently low concentration of plasma cfDNA samples [4,10,17,20,26,72,73], and also the fact that cfDNA originates from every cell undergoing apoptosis or necrosis throughout the whole body, thus diluting the myeloma-specific DNA fragments. We noticed an indication of this latter phenomenon, as during the bioinformatics analyses, we got typically lower ratio of productive immunoglobulin sequences from cfDNA samples (S2 Table). In case of the PBMC samples, although it is shown that MM patients can harbor circulating myeloma cells [14–17], not every patient’s myeloma leaves the bone marrow in a detectable way. Moreover, the tumor cells have a usually low presence in the PBMC fraction of blood, which raises the same issue of sample dilution, as with the cfDNA, however enrichment of circulating tumor cells may improve this limitation [22].

In order to further improve our NGS system, we already started to incorporate the enhanced, standardized and MRD-oriented EuroClonality-NGS primer sets for Ig FR1 heavy chain and Ig light chain [74], simultaneously launching the analysis of a larger and cytogenetically diverse patient cohort with dual aims: testing this new NGS system while validating our results obtained so far.

## Conclusions

In this study our results suggest that monitoring multiple myeloma disease burden by analysis of rearranged immunoglobulin genes is feasible using liquid biopsy procedures, thus indicating the relevance of our V(D)J-based molecular systems in a clinical setting. Early data on a small but well-characterized patient cohort (t(4;14) myeloma) suggests that liquid biopsy results together with other nucleic acid-and cytometry-based methods show good correlation with clinical measures, thus provide the basis for further development of these techniques with the goal of sensitive MRD monitoring.

Limited by the small number of patients enrolled in this study, we could not agree on a conclusively better method for myeloma MRD monitoring, nevertheless we found that in certain situations we can prioritize certain approaches. According to sample availability, we noticed trends towards the possible advantage of NGS analysis if bone marrow sampling is necessary (e.g., for other routine measurements), and the use of ddPCR for monitoring from peripheral blood, especially applied to cfDNA samples. Moreover, we found that immunoglobulin heavy and light chain NGS analyses are complementary approaches and thus it is reasonable to use both methods in parallel. Despite the previously mentioned limitations of this study, based on our results we can conclude that the analysis of liquid biopsy may yield a dynamic measure of the overall myeloma burden, provided the appropriate sample quality.

These findings highlight the potential use of these V(D)J-based molecular techniques in complementing the already implemented routine methods (MFC, IGH-MMSET RT-qPCR) by providing supplementary information on the patients’ disease status, thus potentially helping clinical decision-making. To support our findings, we need additional patient groups with deep and sustained response for validation. Nevertheless, the significant correlations to clinical data indicate a promising trend which must be strengthened by further analyses.

## Supporting information

S1 Fig Representative dot plots of an MFC measurement from patient MM-4, illustrating the overall gating strategy from the m1 bone marrow sample (05.11.2019). (A) Exclusion of duplicate events based on FSC-A and FSC-H. (B) Exclusion of debris based on FSC and SSC. (C) Positive gating of the plasma cells (black, CD138+CD38+). (D) Distinction between the normal (green, CD19+) and malignant (red, CD19-) plasma cells. (E-H) Characterization of the different plasma cell populations (normal: Green, malignant: Red).
(TIF)

S2 Fig Representative dot plots of MFC measurements from patient MM-7, illustrating longitudinal monitoring during this study. Each dot plot represents a measurement shown as the normal (green, CD19+) and the malignant (red, CD19-) plasma cell populations: Sampling timepoints (day-month-year) and sample types are provided for all plots.
(TIF)

S3 Fig Immunoglobulin light chain PCR products of diagnostic bone marrow samples on 1.5% agarose gel. Patient MM-5 with the dominant 300 bp product, and patient MM-6 with a representative PCR product distribution concentrating in the 150–200 bp range. The rest of the patients had similar amplicon distribution as depicted with patient MM-6, even though some of them also had pronounced PCR product presence in the 300 bp region as well, but it was never stronger than the 150–200 bp range. dBM = diagnostic bone marrow sample, L = light chain specific PCR products, MW = molecular weight: GeneRuler 1kb Plus DNA Ladder.
(TIF)

S1 Table Clinical data of all patients enrolled in our study. (XLSX)

S2 Table NGS technical details and sequencing data, as well as the ddPCR technical details and data, both minus the target detection results. (XLSX)

S3 Table The summary of target detection / monitoring results from all methods. (XLSX)

S1 File Additional methodological details and supplementary results. (DOCX)

S1 Raw images (PDF)
