# Supplementary figures and images for: Liquid biopsy-based monitoring of residual disease in multiple myeloma by analysis of the rearranged immunoglobulin genes–A feasibility study
Source: PLoS One. 2023 May 26;18(5):e0285696. doi: 10.1371/journal.pone.0285696 (PMC10218758; doi:10.1371/journal.pone.0285696)

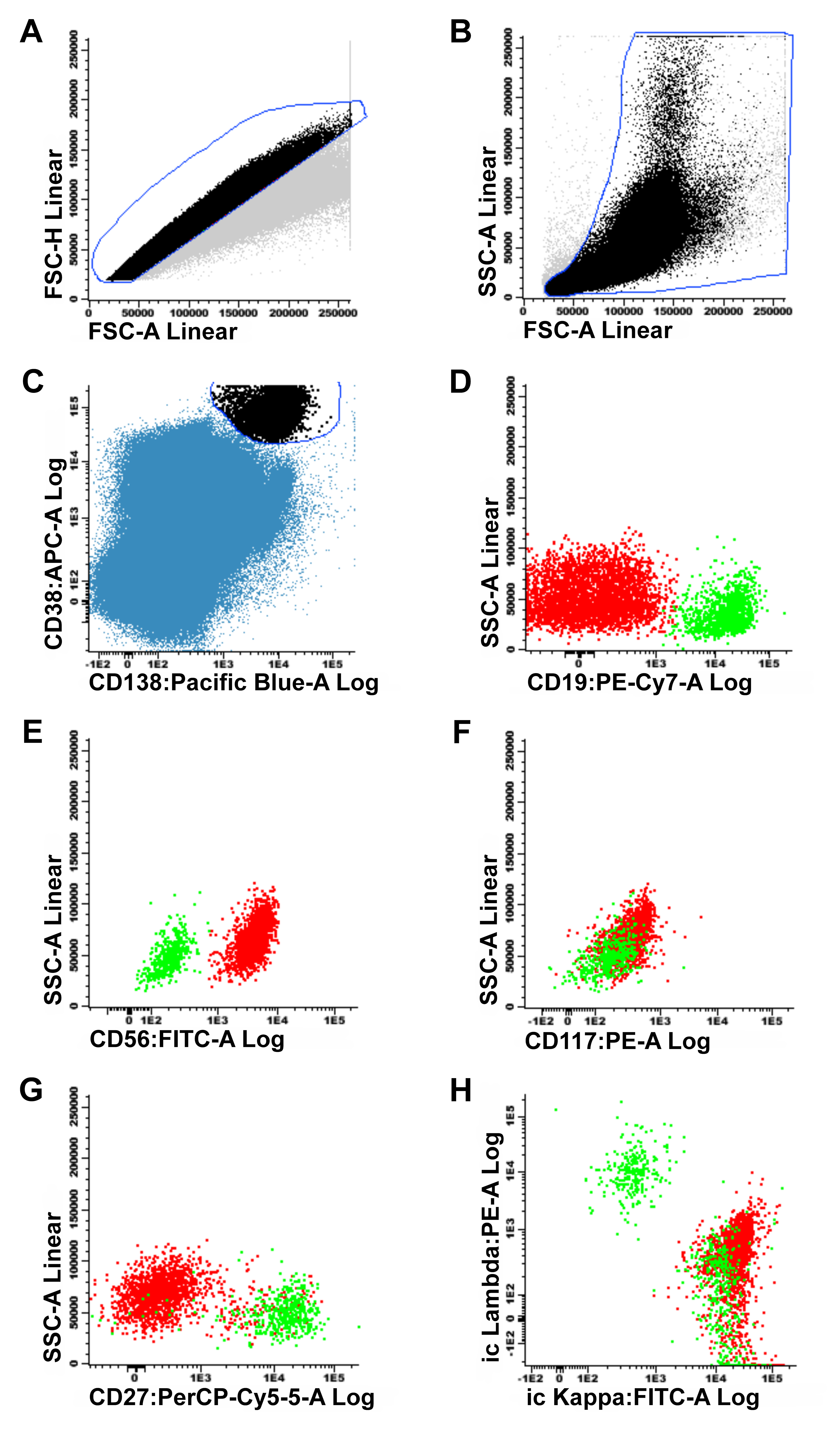

Supplement: S1 Fig — (A) Exclusion of duplicate events based on FSC-A and FSC-H. (B) Exclusion of debris based on FSC and SSC. (C) Positive gating of the plasma cells (black, CD138+CD38+). (D) Distinction between the normal (green, CD19+) and malignant (red, CD19-) plasma cells. (E-H) Characterization of the different plasma cell populations (normal: Green, malignant: Red). (TIF) [file pone.0285696.s001.tif]

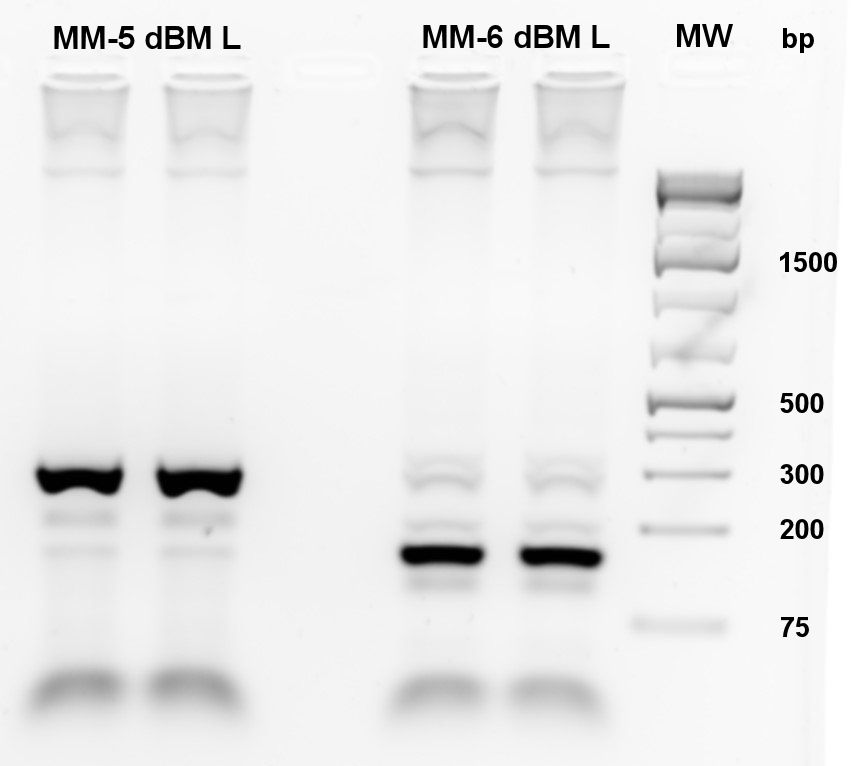

Supplement: S3 Fig — Patient MM-5 with the dominant 300 bp product, and patient MM-6 with a representative PCR product distribution concentrating in the 150–200 bp range. The rest of the patients had similar amplicon distribution as depicted with patient MM-6, even though some of them also had pronounced PCR product presence in the 300 bp region as well, but it was never stronger than the 150–200 bp range. dBM = diagnostic bone marrow sample, L = light chain specific PCR products, MW = molecular weight: GeneRuler 1kb Plus DNA Ladder. (TIF) [file pone.0285696.s003.tif]
