# Supplementary material for: Liquid biopsy-based monitoring of residual disease in multiple myeloma by analysis of the rearranged immunoglobulin genes–A feasibility study
Source: PLoS One. 2023 May 26;18(5):e0285696. doi: 10.1371/journal.pone.0285696 (PMC10218758; doi:10.1371/journal.pone.0285696)
Supplement: S1 File — (DOCX) [file pone.0285696.s007.docx]

***Supporting Information***

**Methods**

**DNA isolation for Ig V(D)J-based analyses**

For gDNA isolation, a few modifications applied to the DNeasy Blood&Tissue Kit (Qiagen) ‘Animal Blood or Cells – 1/C’ protocol were the following: cell lysis for 1 hour at 56°C with 1400 rpm shaking; centrifugation with the AW2 washing buffer for 1 minute at 11 000xg; drying the membrane by centrifugation of the empty tube for 3 minutes at 20 000xg; incubation of the membrane with the elution buffer for 4 minutes at room temperature before final centrifugation. The elution process was done according to the high-yield protocol of the kit.

In case of cfDNA isolation, slight modifications to the QIAamp Circulating Nucleic Acid Kit (Qiagen) protocols (’Purification of Circulating Nucleic Acids from 1 ml, 2 ml or 3 ml Serum or Plasma’ and ’Purification of Circulating Nucleic Acids from 4 ml or 5 ml Serum or Plasma’) were the following: separate addition of Buffer ACL and Carrier RNA, using more Carrier RNA per sample (1.1 µl).

**Multiplex PCRs targeting the immunoglobulin genes**

The pre-PCR steps were carried out in a PCR cabinet (UVT-B-AR DNA/RNA UV-Cleaner Box, BioSan), all the surfaces were treated with DNA ExitusPlus IF decontaminant solution (PanReac AppliChem) a priori.

Concerning the template DNA amounts, 500 ng of gDNA and 25 ng of cfDNA were used when possible, but for low concentration samples, less template was used to avoid reaction inhibition by carry-over salts in the buffer and total depletion of the samples.

The PCR conditions for one reaction were the following: 50 µl final volume, 10 mM each dNTPs (Thermo Scientific), 2 pmol/µl each forward FR1 primer mix or forward κ+λ primer mix (custom order, Integrated DNA Technologies), 10 pmol/µl reverse J_H_ primer or reverse κ+λ primer mix (custom order, Integrated DNA Technologies), 0.8 units Phusion High-Fidelity DNA polymerase (Thermo Scientific).

The touchdown heat profiles of the PCRs were the following. For the heavy chain: initial denaturation at 98°C for 2 minutes; 32 cycles of denaturation at 98°C for 15 seconds, annealing starting at 67°C and decreasing gradually in each cycle until 57°C for 30 seconds, extension at 72°C for 30 seconds; final extension at 72°C for 5 minutes. The light chain reaction had 30 cycles with the annealing temperature decreasing from 65°C to 55°C. Both the heavy and the light chain programs had 45 cycles for the cfDNA samples, due to their inherently lower DNA concentration.

The BIOMED-2 heavy chain FR1 specific primer set consists of 6 forward primers and a reverse primer. The BIOMED-2 light chain kappa and lambda specific primer sets consist of 6+2 forward primers (kappa and lambda, respectively), and 2+1 reverse primers (kappa and lambda, respectively) [44]. For our experiments, a 1:1 mixture of the corresponding primers was used for the forward and reverse primer mixes.

**Agarose gel electrophoresis and PCR product extraction**

1.5% agarose gel was prepared with SeaKem LE Agarose (Lonza), freshly made 1xTBE buffer (Tris-borate-EDTA buffer) and was pre-stained with GelRed Nucleic Acid Stain (10 000 x, in water, Biotium).

The samples were prepared for electrophoresis with 6x DNA Loading Dye (Thermo Scientific), and 2 µl of GeneRuler 1kb Plus DNA Ladder (Thermo Scientific) was used as molecular weight marker. The conditions of the electrophoresis were the following: 120 V for 40 minutes in case of the heavy chain samples and 60 minutes for the light chain samples, by using a Gelco GH330 horizontal gel electrophoresis system and a Bio-Rad Wide Mini-Sub Cell GT horizontal gel electrophoresis system, together with a GN344 power supply (Biostep). The results were checked in a SynGene G:BOX Chemi XRQ gel documentation system with a GX-2020M-E transilluminator (302 nm) using the Manual Capture mode with 200-250 ms exposition times.

In order to avoid cross-sample contamination, we used DNA ExitusPlus IF decontaminant solution (PanReac AppliChem) before assembling the electrophoresis system. While loading the samples onto the gel, we left one slot empty between them. The extraction of the appropriate PCR products was carried out with single-use Gel Cutting Pipette Tips (Axygen), aided with a clean scalpel if necessary.

**Library construction and next-generation sequencing**

The library quantity and quality/fragment distribution were checked on a Qubit fluorometer (Thermo Sceintific) and a Bioanalyzer 2100 (Agilent), respectively. The libraries were diluted to 4 nM universally and the correct pooling of the samples was calculated. The final loading concentration of the pooled libraries was set to 8 pM. The sequencing was carried out with the MiSeq Reagent Kit v2 (Illumina) 2x250bp kit for the heavy chain libraries (and the 300 bp light chain libraries) and the 2x150bp kit for the 150-200 bp light chain libraries.

In order to avoid contamination of the monitoring/follow-up samples from supposedly target-rich diagnostic samples, each patients’ diagnostic bone marrow sample was sequenced in separate runs from their monitoring samples.

The majority of the diagnostic bone marrow samples were sequenced from a diluted library as we hypothesized that those repertoires contain high percentage of the target, thus lower coverage will still be necessary for target identification. In turn, a bit more coverage can be obtained for the monitoring samples on the sequencing flow cell in the same run.

**ddPCR protocol**

**Assay design to IgH-CDR3**

To achieve highly specific assays, we targeted the CDR3 of heavy chain myeloma clone sequences (obtained from the NGS analyses), specifically the N/P nucleotide parts identified by the IMGT/V-QUEST tool [75-77]. The design principle was either the primers or the probe should overlap these N/P nucleotides, for which the Primer3Plus assay design tool [78] was used with a premade “Settings file” specified for ddPCR assays, provided by the Bio-Rad customer support. The custom assay sequences can be found below in Table A. For these IgH-CDR3 specific assays, 6-FAM (6-carboxyfluorescein) fluorophore with Iowa Black FQ quencher was used on the probes (PrimePCR Custom Assay, Bio-Rad). To make our ddPCR analyses more robust and reliable, an *RPP30* assay (dHsaCP1000485, Bio-Rad), targeting the housekeeping *RPP30* (ribonuclease P/MRP subunit p30) gene, was used as a reference assay in multiplex setting, thus the probe was labelled with HEX (hexachlorofluorescein) fluorophore and Iowa Black FQ quencher.

**Table A. Patient immunoglobulin heavy chain CDR3-specific custom ddPCR assay sequences.**

| **Assays** | **Sequences** |
| --- | --- |
| 123-2012_H for patient MM-1 | |
| Forward primer | 5’- AGACATTCCCAAGAACACACT -3’ |
| Reverse primer | 5’- GGATGATCACTCCTTCCATCC -3’ |
| Probe with 5’ 6-FAM | 5’- AACAACCTGAGAGGCGAGG -3’ |
| 155_H for patient MM-2 | |
| Forward primer | 5’- GCCTGAGATCTGACGACAC -3’ |
| Reverse primer | 5’- CTTACCTGAGGAGACGGTGA -3’ |
| Probe with 5’ 6-FAM | 5’- ACGACGGAGACTACTACGGT -3’ |
| 196_H for patient MM-3 | |
| Forward primer | 5’- TCTGCAAATGAACAGCCTGA -3’ |
| Reverse primer | 5’- CGAAGGATAAATGACTCGCAC -3’ |
| Probe with 5’ 6-FAM | 5’- CCGAGGACACGGCTGTATAT -3’ |
| 218_H for patient MM-4 | |
| Forward primer | 5’- ATGCTGACATCTGACGACAC -3’ |
| Reverse primer | 5’- CTGTTACCATAGTCCCTTGGC -3’ |
| Probe with 5’ 6-FAM | 5’- AGACTTGGGCAATAAGGCGT -3’ |
| 226_H for patient MM-5 | |
| Forward primer | 5’- AGAACCGCCTACCTCCAG -3’ |
| Probe with 5’ 6-FAM | 5’- CAAGATGTGGAAAACTCCGC -3’ |
| Probe with FAM | 5’- CTCGGACACCGCCATGTATT -3’ |
| 227_H for patient MM-6 | |
| Forward primer | 5’- GACTTGAGCAGCCTGACATC -3’ |
| Reverse primer | 5’- AGACGGTGACCAGGGTTC -3’ |
| Probe with 5’ 6-FAM | 5’- AAGAACTACTACTTTGACTCCTGGG -3’ |
| 229_H for patient MM-7 | |
| Forward primer | 5’- ACAACGGCAAGAACTCACTG -3’ |
| Reverse primer | 5’- CTAATCCAGGACCGCTCGTA -3’ |
| Probe with 5’ 6-FAM | 5’- AGCCTTAAACCCGAGGACAC -3’ |
| 294_H for patient MM-8 | |
| Forward primer | 5’- CCAGGTGGTCCTTACAATGG -3’ |
| Reverse primer | 5’- CTCAGCTTCTCTATCCGTGC -3’ |
| Probe with 5’ 6-FAM | 5’- ACACCGCCACCTTTTACTGT -3’ |

**ddPCR measurements**

The pre-PCR steps were done in a PCR cabinet (UVT-B-AR DNA/RNA UV-Cleaner Box, Biosan), with the following reagents for one reaction: 11 µl ddPCR Supermix for Probes (No dUTP), 1 µl IgH-CDR3 target specific FAM assay, 1 µl *RPP30* HEX assay (Bio-Rad). 12 µl of the mixes were aliquoted into the appropriate positions of a 96-well PCR plate (Bio-Rad). When possible, 25 ng of template DNA (in a total volume of 10 µl) was added to the corresponding wells resulting in a final reaction volume of 22 µl/well prior to droplet generation. For the PCR, the following heat profile was applied: enzyme activation at 95°C for 10 minutes; 40 cycles of denaturation at 94°C for 30 seconds, annealing at 60°C for 1 minute; final enzyme inactivation at 98°C for 10 minutes.

**IGH-MMSET RT-qPCR protocol**

The qPCR program for *IgH::MMSET* mRNA detection started with an initial denaturation at 95˚C for 10 min, 50 cycles of denaturation at 95˚C for 15 sec, annealing at 63˚C for 60 sec followed by the extension step at 72˚C for 60 sec.

During initial optimization of the IGH-MMSET RT-qPCR method, serial dilutions were measured from both the fusion mRNA target and the housekeeping gene *ABL1* mRNA. As both systems had nearly identical efficiencies, the deltaCt method could be used for the calculation of the fusion mRNA target percentages.

**Number of the analyzed follow-up samples**

We summarized the number of the analyzed follow-up samples from each patient in Table B, and the total number of follow-up samples from all patients analyzed by each method in Table C.

**Table B. The number of monitoring timepoints and follow-up samples analyzed from each patient.**

| **Patient code** | **Nb. follow-up timepoints** | **Nb. follow-up samples (total)** |
| --- | --- | --- |
| MM-1 | 3 | 7 |
| MM-2 | 5 | 14 |
| MM-3 | 1 | 3 |
| MM-4 | 6 | 13 |
| MM-5 | 5 | 10 |
| MM-6 | 4 | 9 |
| MM-7 | 4 | 10 |
| MM-8 | 4 | 9 |

**Table C. The number of total follow-up samples analyzed by each method.**

| **Methods** | **Nb. follow-up samples (total)** |
| --- | --- |
| IGH-MMSET qPCR | 13 |
| MFC | 15 |
| NGS-H | 69 |
| ddPCR | 76 |
| NGS-L | 60 |

**Methods for the analysis of the samples from patient MM-4**

Extracted and purified amplicons of unusual sizes (around 900 bp and 1200 bp for heavy and light chain, respectively) were subjected to Sanger sequencing. Preparatory steps were done by using the CloneJET PCR Cloning Kit (Thermo Scientific) following the Blunt-End Cloning Protocol. Competent Escherichia coli WK6 cells were transformed with the constructs containing the PCR products by standard calcium chloride protocol and spread on LB agar plates containing 100 µg/ml ampicillin. Colonies were picked randomly and tested via colony PCR for the presence of the IgH or IgL amplicon using the protocol provided by the CloneJET kit. As primers, the pJET1.2 forward sequencing primer specific to the vector and the appropriate reverse BIOMED-2 primer(s) targeting the insert were used. Five of the positive colonies were collected and cultured overnight in 5 ml LB medium containing 100 µg/ml ampicillin, for plasmid DNA purification using the ZymoPURE Plasmid Miniprep Kit (Zymo Research), following the manufacturer’s protocol. These plasmids were sequenced with the Sanger method by Biomi Kft. (Gödöllő, Hungary), using both the forward and the reverse sequencing primers from the CloneJET kit. The sequences were analyzed by first creating a consensus sequence from the forward and reverse reads, then using the IMGT/V-QUEST Tool [75-77] for immunologically relevant evaluation and screening for the IgH or IgL amplicons.

**Results**

**DNA isolation**

The cfDNA yield of the plasma samples was 3-4 ng/µl in average (ranging from 0.1 ng/µl to 10 ng/µl), which accounted for a total yield of 200 ng in average (in the range of 5-500 ng).

The gDNA yield of bone marrow and PBMC samples was 123 ng/µl in average (between 94 ng/µl and 175 ng/µl), providing us a total gDNA of 24.6 µg in average (ranging from 18.8 µg to 35 µg).

**Patients’ V and J genes and alleles**

The complete information on the identified patient-specific immunoglobulin sequences is summarized in Table D.

**Table D. Complete list of the V and J genes and alleles identified from each patient at diagnosis. Data from the IMGT/HighV-QUEST analyses.**

| **Patient code** | **V gene and allele** | **J gene and allele** |
| --- | --- | --- |
| MM-1 | Homsap *IGHV3-23*01 F* or **04 F* or *D*01 F* | Homsap *IGHJ4*02 F* |
|  | Homsap *IGKV1-5*01 F* or **02 F* or **03 F* | Homsap *IGKJ1*01 F* or Homsap *IGKJ4*01 F* or **02 F* |
| MM-2 | Homsap *IGHV1-18*01 F* or **04 F* | Homsap *IGHJ6*02 F* |
|  | Homsap *IGLV2-14*01 F* or **02 F* or **03 F* or **04 F* or Homsap *IGLV2-18*02 F* | Homsap *IGLJ2*01 F* or Homsap *IGLJ3*01 F* or **02 F* |
| MM-3 | Homsap *IGHV3-21*01 F* or **02 F* | Homsap *IGHJ6*-02 F* |
|  | Homsap *IGKV3-20*01 F* or *3D-20*01 F* | Homsap *IGKJ2*01 F* |
| MM-4 | Homsap *IGHV1-2*02 F* | Homsap *IGHJ3*01 F* |
| MM-5 | Homsap *IGHV5-51*01 F* or **02 F* or **03 F* or **07 F* | Homsap *IGHJ3*02 F* |
|  | Homsap *IGKV2-30*01 F* or **02 [F]* or *2D-30*01 F* | Homsap *IGKJ2*01 F* |
| MM-6 | Homsap *IGHV1-69-*06 F* or **14 F* | Homsap *IGHJ4*02 F* |
|  | Homsap *IGKV1-5*01 F* or **02 F* or **03 F* | Homsap *IGKJ4*01 F* |
| MM-7 | Homsap *IGHV3-21*01 F* or **02 F* or Homsap *IGHV3-21*05 (F)* | Homsap *IGHJ3*02 F* |
|  | Homsap *IGLV3-25*03 F* | Homsap *IGLJ2*01 F* or Homsap *IGLJ3*01 F* |
| MM-8 | Homsap *IGHV2-70*01 F* or **10 F* or **11 F* or **12 F* or **15 F* or **18 F* | Homsap *IGHJ4*02 F* |
|  | Homsap *IGKV1-39*01 F* or *1D-39*01 F* | Homsap *IGKJ2*01 F* |

**Myeloma targets of patients MM-241 and MM-249 (heavy chain)**

We identified the myeloma heavy chain targets of the two patients who were excluded from further analysis due to their early passing and hence the lack of follow-up samples. The CDR3 amino acid (aa) sequences and the possible genes and alleles are shown in Table E.

**Table E. The heavy chain CDR3 amino acid (aa) sequences and the V and J genes and alleles identified from patients MM-241 and MM-249 at diagnosis. Data from the IMGT/HighV-QUEST analysis.**

| **Patient code** | **CDR3 sequence (aa)** | **V_H_ gene and allele** | **J_H_ gene and allele** |
| --- | --- | --- | --- |
| MM-241 | CATFPFSAFSSGDYYFDQW | Homsap *IGHV3-23*01 F*, or *IGHV3-23*04 F* or *IGHV3-23D*01 F* | Homsap *IGHJ4*02 F* |
| MM-249 | CAHISGESVPGGVYLYYGMDVW | Homsap *IGHV2-5*04 F* | Homsap *IGHJ6*02 F* |

**Results from the analysis of the samples from patient MM-4**

Patient MM-4 had a unique diagnostic bone marrow PCR pattern in both the immunoglobulin heavy and the light chain reactions. The usual size ranges contained no clonal sequences, but a great abundance of PCR products was present around 900 bp and 1200 bp in the heavy and the light chain samples, respectively. The heavy chain amplicons’ analysis by Sanger sequencing showed the presence of a clonal, productive immunoglobulin sequence which was suggested to be disease-specific of this patient by further analyses. We found the presence of that unusual PCR product in certain monitoring samples, like the m1 bone marrow or the m2 and m3 cfDNA samples, where we found that the strength of this band coincided with the disease intensity. That said, in the m1 bone marrow sample it was much weaker, while in both the m2 and m3 cfDNA samples it became more pronounced again, mirroring the temporary retraction of the disease before the relapse. These products were also analyzed by the same method as the diagnostic bone marrow, confirming the presence of the same clonal sequence throughout the disease course. Of note, the monitoring of the normal PCR product range with NGS showed the same trend in target detection, also strengthened by the ddPCR analyses. In case of the light chain analysis, the same methods yielded no productive, rearranged immunoglobulin sequence from the diagnostic bone marrow, hence the lack of the light chain monitoring data.

**Representative dot plots from the MFC measurements**

We have chosen two representative patients for the illustration of our MFC results. On the S1 Figure, the gating strategy is shown from a bone marrow sample of patient MM-4 as an example. S2 Figure shows results of longitudinal monitoring with patient MM-7 as a representative example.

**Gel image of light chain PCR products**

In order to illustrate the case of patient MM-5’s diagnostic bone marrow sample light chain PCR results (presented in the Results section of the main text), a supporting gel image was prepared as the S3 Fig, showing the sample in question as well as a representative sample (MM-6’s) of the other patients for reference.

**Decoding patient IDs for the Sequence Read Archive (SRA) entry**

The original anonymous patient IDs were modified in our paper for the ease of reading, but as the FASTQ sequences from the NGS runs were initially uploaded to the SRA under these original anonymous IDs, here we provide Table F to help the decoding of our patients’ raw NGS data. The 3 patients excluded from analyses retained their original anonymous IDs wherever they appear in the paper and the supporting information files.

Of note, for the NGS Sample IDs in S2 Table and S3 Table we also used the original anonymous patient IDs (while clearly indicating the updated patient codes) in order to facilitate the comparison to the raw files stored in our SRA entry.

**Table F. The patient codes used in the paper and their corresponding original anonymous IDs used in the SRA submission (**[**PRJNA880686**](https://www.ncbi.nlm.nih.gov/sra/PRJNA880686)**).**

| **Patient code in paper** | **Original anonymous ID (SRA)** |
| --- | --- |
| MM-1 | MM-123 |
| MM-2 | MM-155 |
| MM-3 | MM-196 |
| MM-4 | MM-218 |
| MM-5 | MM-226 |
| MM-6 | MM-227 |
| MM-7 | MM-229 |
| MM-8 | MM-294 |

**References**

1. van Dongen JJM, Langerak AW, Brüggemann M, Evans PAS, Hummel M, Lavender FL, et al. Design and Standardization of PCR Primers and Protocols for Detection of Clonal Immunoglobulin and T-Cell Receptor Gene Recombinations in Suspect Lymphoproliferations: Report of the BIOMED-2 Concerted Action BMH4-CT98-3936. Leukemia 2003, 17 (12), 2257–2317. [https://doi.org/10.1038/sj.leu.2403202.](https://doi.org/10.1038/sj.leu.2403202)
2. IMGT/V-QUEST. Available online: <https://www.imgt.org/IMGT_vquest/input> (accessed on 9 August 2022)
3. Brochet X, Lefranc MP, Giudicelli V. IMGT/V-QUEST: The Highly Customized and Integrated System for IG and TR Standardized V-J and V-D-J Sequence Analysis. Nucleic Acids Res 2008, 36 (Web Server issue), W503-508. [https://doi.org/10.1093/nar/gkn316.](https://doi.org/10.1093/nar/gkn316)
4. Giudicelli V, Brochet X, Lefranc MP. IMGT/V-QUEST: IMGT Standardized Analysis of the Immunoglobulin (IG) and T Cell Receptor (TR) Nucleotide Sequences. Cold Spring Harb Protoc 2011, 2011 (6), 695–715. [https://doi.org/10.1101/pdb.prot5633.](https://doi.org/10.1101/pdb.prot5633)
5. Primer3Plus. Available online: <https://www.bioinformatics.nl/cgi-bin/primer3plus/primer3plus.cgi> (accessed on 9 August 2022)
