# Supplementary material for: Liquid biopsy-based monitoring of residual disease in multiple myeloma by analysis of the rearranged immunoglobulin genes–A feasibility study
Source: PLoS One. 2023 May 26;18(5):e0285696. doi: 10.1371/journal.pone.0285696 (PMC10218758; doi:10.1371/journal.pone.0285696)
Supplement: S1 Raw images — (PDF) [file pone.0285696.s008.pdf]

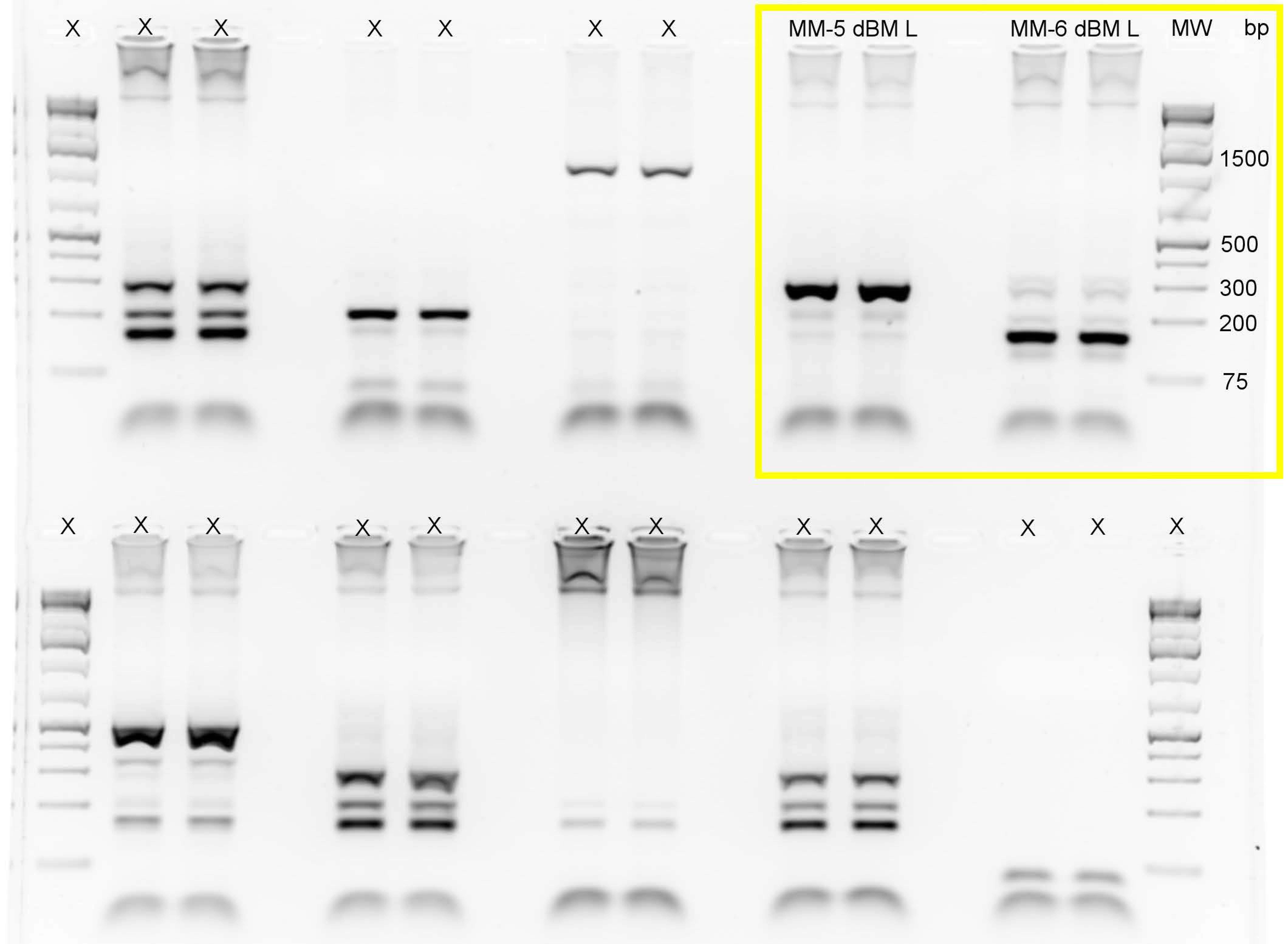

<u>Image capturing method:</u>
G:Box Chemi XRQ (SynGene) gel documentation system
GX-2020M-E transilluminator (302 nm)
250 ms exposition time

Part of the image used for S3 Fig is indicated with a yellow rectangle.
